# Chemical Profiling and Cosmetic Potential from *Anacardium humile* and *Anacardium occidentale*

**DOI:** 10.1021/acsomega.5c12447

**Published:** 2026-03-19

**Authors:** Carlos F. da Silva, Noelle C. F. dos Santos, Isabella M. Do Nascimento, Cleysla M. da Silva, Patricia S. Lopes, Larissa S. Costa, Lorena R. F. de Sousa, Adriana P. M. G. Macedo, Felipe L. Coelho, Carlos André F. Moraes, Maico R. Severino, Vanessa G. Pasqualotto Severino

**Affiliations:** † Chemistry Institute, Federal University of Goiás, Esperança Avenue, Goiânia, Goiás 74690-900, Brazil; ‡ Institute of Environmental, Chemical and Pharmaceutical Sciences, Department of Pharmaceutical Sciences, Federal University of São Paulo, Diadema, São Paulo 09913-030, Brazil; § Chemistry Institute, Federal University of Catalão, Catalão, Goiás 75704-020, Brazil; ∥ Chemistry Institute, Federal University of São Carlos, São Carlos, São Paulo 13565-905, Brazil; ⊥ Faculty of Sciences and Technology, Federal University of Goiás, Aparecida de Goiânia, Goiás 74971-451, Brazil

## Abstract

*Anacardium humile* St. Hil. and *A. occidentale* L. are Brazilian species of nutritional and economic importance, but their pseudofruits—especially *A. humile*—are underexplored. This study chemically profiles ethanolic extracts from mixed pseudofruits of both species, collected from two Goiás regions, and evaluates their cosmeceutical potential. Chemical profiling was primarily performed by HPLC-ESI-QTOF-MS/MS combined with GNPS molecular networking, yielding putative annotations, while ^1^H NMR provided complementary chemical fingerprinting. Nine compounds were annotated by LC-MS/MS, mainly glycosylated flavonoids (quercetin and myricetin derivatives), together with a disaccharide and an anacardic acid. In addition, the ^1^H NMR spectra of the ethyl acetate fractions showed signals that support the presence of *p*-coumaric acid. The crude extracts showed low cytotoxicity in HaCaT cells (IC_50_ = 41.330 ± 1.618 mg/mL for AHB, 0.891 ± 0.079 mg/mL for AHK), with additional PrestoBlue data supporting safety. Enzyme assays revealed moderate elastase inhibition (up to 48.4 ± 4.0% at 100 μg/mL) and low tyrosinase inhibition (≈9%). The extracts also exhibited strong UV absorption (<300 nm) and photostability after UV exposure (254 and 363 nm for 8 h). These results suggest that *Anacardium* pseudofruits, with their flavonoid, hydroxycinnamate, and carotenoid profiles, are promising for antiaging/photoprotective applications.

## Graphical abstract

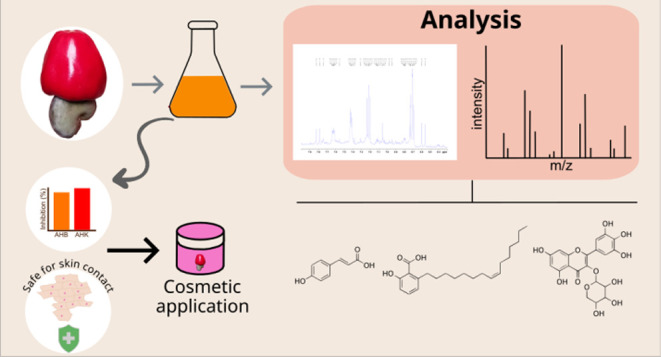

## Introduction

*Anacardium humile* A.St.-Hil. (Anacardiaceae), popularly known as “cajuzinho-do-cerrado” or “cajuí”, is an endemic Brazilian species predominantly occurring in the Cerrado savanna (*sensu stricto*).^1^ Within the genus, *Anacardium occidentale* L. (cashew) shows broader distribution and more extensive use.^2^ Both species have a long history in traditional medicine (bark and leaves) to manage diarrhea, skin lesions, ulcers, gastritis, among other conditions.^3,4^ To our knowledge, the pseudofruit pulp of *A. humile* has not been comprehensively profiled in conjunction with HaCaT 3D safety and enzyme-target assays, which adds novelty and translational value to the present investigation.

The fruits (nuts) and pseudofruits (pulp) of *A. humile* and *A. occidentale* are also nutritionally relevant. Their pseudofruits display yellow to red hues—pigments with potential industrial interest—are acidic in flavor, and are consumed fresh or used in juices, sweets, and jams,^5–7^ contributing to food security and income generation, with implications for natural food colorants and value-added uses.

Chemical studies in *A. humile* have reported terpenoids, steroids, tannins, anacardic acids in leaves,^7,8^ and vitamin C, β-carotene,^9^ and polyphenols (including catechin and amentoflavone) in the pseudofruit,^3^ along with other compounds found in both leaves and pseudofruits.^10^ For *A. occidentale*, cardols, cardanols, and anacardic acids occur in nuts and nut liquid,^11^ while pseudofruits are rich in phenolic compounds (e.g., *p*-coumaric acid), flavonoids (e.g., astragalin), anthocyanins (e.g., petunidin), catechins/derivatives,^12^ and carotenoid esters.^13^

Despite these advances, the pseudofruit pulp of *A. humile* remains comparatively underexplored, and the linkage between its composition and functional end points of cosmetic interest is still limited. Meanwhile, the growing demand for natural and sustainable ingredients in skincare underscores the need for chemical profiling and safety/efficacy evaluation. In this context, elastase and tyrosinase—key enzymes involved in skin aging and pigmentation—are relevant targets for antiaging and skin-brightening applications.^14–18^ Mixed pseudofruits were selected to reflect practical processing and to capture compositional variability across sourcing regions.

The objective of this study was to chemically profile ethanolic extracts from mixed pseudofruits of *A. humile* and *A. occidentale* and to evaluate their safety and potential for cosmetic applications. Chemical profiling was based on HPLC-ESI-QTOF-MS/MS compound annotation using GNPS molecular networking, complemented by ^1^H NMR analysis to describe the overall chemical features of the extracts and fractions. Safety and cosmetic-related bioactivities were evaluated through cytotoxicity assays in HaCaT keratinocytes (monolayer and 3D spheroids) and inhibitory activity against elastase and tyrosinase.

## Materials and Methods

### Sample Collection and Preparation

Approximately 5 kg of mixed cashew pseudofruits were collected from two regions in northeastern Goiás, Brazil: Brazlândia and the Kalunga community. The samples were coded as AHB (Brazlândia) and AHK (Kalunga) and used as reference throughout the study. Collections took place between September and November 2021. Species identification, based on voucher specimens examined by Prof. Dr. Rafael B. Pinto, confirmed that both *A. humile* and *A. occidentale* were present in the collected material. The vouchers were deposited in the herbarium of the Federal University of Goiás under accession numbers UFG 43002 (*A. humile*) and 50524 (*A. occidentale*). Access to genetic resources was registered in the National System for the Management of Genetic Heritage and Associated Traditional Knowledge (SISGEN) under code AC7A261.

An aliquot of 2 kg from each batch of pseudofruits was sliced and lyophilized for 48 h using a Liotop L18 freeze-dryer. The lyophilized material was ground using a blender, vacuum-sealed in plastic bags, and stored under refrigeration until analysis.

### Extraction

Secondary metabolites were initially extracted from 5.0 g of lyophilized material using 50 mL of absolute ethanol, assisted by an ultrasonic bath at 45 °C for 60 min. To improve extraction efficiency, five successive extractions were performed for each sample under identical conditions. The combined extracts were concentrated under reduced pressure at 40 °C, transferred to glass vials, and air-dried to constant mass, yielding the crude ethanolic extracts, which were stored under refrigeration until further use.

Subsequently, an aliquot of 2 g of each crude ethanolic extract was redissolved in 100 mL of a methanol:water solution (1:2, v/v) and subjected to sequential liquid–liquid partitioning. The hydromethanolic solution was transferred to 250 mL separatory funnels and exhaustively extracted five times with 25 mL of hexane, followed by five extractions with 25 mL of ethyl acetate. The remaining hydromethanolic phase constituted the polar fraction. This procedure afforded the hexane (Hex), ethyl acetate (Ac), and hydromethanolic (Aq) fractions, which were individually concentrated under reduced pressure and transferred to glass vials for final drying. Both the crude ethanolic extracts and the resulting fractions (Hex, Ac, and Aq) were subsequently used in the chemical and biological analyses.

### Extract Analysis

#### ^1^H NMR Spectroscopy

Approximately 15 mg of each sample (crude extracts and fractions) were analyzed on a Bruker Avance Neo spectrometer (9.4 T; 400 MHz for ^1^H). Deuterated methanol (MeOD-*d*_4_) was used for AHB, AHBAc, AHBAq, AHK, AHKAc, and AHKAq, and deuterated chloroform (CDCl_3_) for AHBHex and AHKHex. Spectra were manually phased, baseline-corrected, and calibrated (δ 0.00) using tetramethylsilane (TMS); processing was performed in TopSpin 4.2.0 (Bruker, Germany). Deuterated solvents were purchased from Sigma-Aldrich (St. Louis, MO, USA) and Cambridge Isotope Laboratories, Inc. (Tewksbury, MA, USA).

#### High-Performance Liquid Chromatography Coupled to Electrospray Ionization Quadrupole Time-of-Flight Tandem Mass Spectrometry (HPLC-ESI-QTOF-MS/MS)

HPLC-ESI-QTOF-MS/MS analyses were performed on the crude ethanolic extracts to profile polar and midpolar secondary metabolites. Analyses were conducted in both positive and negative ionization modes, and compound annotations were reported as putative (MSI level 2), without confirmation using authentic standards.

Metabolomic profiling was conducted following Brito et al. (2021),^19^ with minor adaptations. Analyses were carried out on an Agilent 1290 Infinity II LC system coupled to a G6545B Q-TOF mass spectrometer equipped with an electrospray ionization (ESI) source.

Chromatographic separation used an Agilent Zorbax XDB-C18 column (2.1 × 100 mm; 1.8 μm; Agilent Technologies, St. Clara, CA, USA), maintained at 33 °C. Injection volume was 3.0 μL and flow rate 0.300 mL/min. The mobile phases were acetonitrile (ACN) + 0.1% formic acid (HCO_2_H) and water +0.1% formic acid and were delivered under the following gradient: 90% ACN from 0–12 min, 100% ACN from 12–17 min, and 5% ACN from 17–22 min.

Mass spectrometric detection was performed in full-scan mode over 22 min (*m*/*z* 150–1,500) in both ESI­(+) and ESI(−). Source settings were: drying gas 10 L/min at 300 °C, nebulizer 35 psig, sheath gas 11 L/min at 350 °C, capillary voltages +3.1/–3.1 kV. MS/MS acquisition used data-dependent selection of the five most intense precursors per cycle, with collision energies ramped from 10 to 59 eV based on precursor *m*/*z* and charge state. Lock-mass calibration ensured mass accuracy within ±5 ppm; data processing used 5 ppm mass tolerance for feature finding and library matching.

#### Molecular Network Analysis

The molecular network was organized into two groups according to sampling location: Group 1, ethanolic extract of pseudofruits from Brazlândia (AHB), and Group 2, ethanolic extract of pseudofruits from the Kalunga community (AHK). Network parameters included precursor-ion and MS/MS fragment mass tolerances of 0.02 Da, cosine similarity >0.6, and a minimum of four matched peaks. Spectral data were matched against the GNPS library,^20^ and fragmentation patterns were proposed for each compound. All compound annotations were assigned a Metabolomics Standards Initiative (MSI) level 2 confidence.

Data processing comprised chromatograms and mass-spectra inspection in MassHunter Qualitative Analysis Navigator (v. B.08.00, Agilent), conversion to .mzML using MSConvert, and feature detection/alignment in MZmine3 (v. 3.9.0). Processed data were submitted to GNPS (via WinSCP, v. 6.3.4), and networks were visualized/edited in Cytoscape (v. 3.10.2).^21^

Annotations were manually validated by examining characteristic MS/MS fragmentation patterns. GNPS result pages are available at: https://gnps.ucsd.edu/ProteoSAFe/result.jsp?task=b726f34d76df439186716554d1bcf47b&view=view_all_annotations_DB

https://gnps.ucsd.edu/ProteoSAFe/result.jsp?task=a11c213510e44b5787a45b4f2a769204&view=view_all_annotations_DB.

#### Thermal Stability

Crude extracts were analyzed by thermogravimetric analysis (TGA-DTG) to evaluate thermal stability. Experiments were carried out on a DTG 60/60H (SHIMADZU), following Mothé and Freitas (2014).^22^ Samples were heated from 30 to 800 °C at 10 °C/min under a synthetic oxidizing atmosphere (gas flow 20 mL/min). Approximately 6 mg of each sample was placed in alumina crucibles for analysis, and a blank run (no sample) was recorded under identical conditions.

Derivative thermogravimetric (DTG) curves were generated from the data using OriginPro 8.5.

#### Photochemical Study

Given the intense coloration of the crude extracts (AHB and AHK), a photochemical study was performed to evaluate color behavior under different conditions. Stock solutions were first prepared at 2400 mg/mL; 50 μL aliquots were then diluted in 950 μL of methanol, followed by addition of 3 mL of distilled water to obtain methanol:water (1:3, v/v) solutions at a final concentration of 30 mg/mL.

Two factors were evaluated: temperature (−10 and 24 °C) and UV exposure. For temperature, solutions were cooled in a salt-ethanol ice bath to −10 °C or equilibrated at 24 °C (laboratory ambient). For UV exposure, solutions were irradiated inside a TLC dark chamber equipped with 254 and 363 nm UV lamps for 8 h.

Measurements were acquired in quartz cuvettes (1 cm path length) using a BEL UV-M51 single-beam spectrophotometer over 190–800 at 2 nm intervals (UV–vis range). The methanol:water (1:3, v/v) mixture served as the blank. Spectra were normalized and plotted in OriginPro 8.5.

### Biological Activity

#### Cytotoxic Activity in Monolayer

Cytotoxicity of the extracts was evaluated following the protocol established by the Organization for Economic Co-operation and Development.^23^ HaCaT cells were cultured at 37 °C, 5% CO_2_, and 97% relative humidity until ∼80% confluence. Depending on solubility, extracts were dissolved in DMSO or ethanol and diluted into Dulbecco’s Modified Eagle Medium (DMEM; Gibco) supplemented with 10% (v/v) fetal bovine serum (FBS, heat-inactivated; LGC Biotechnology), 1% (v/v) antibiotic-antimycotic (10,000 IU/mL penicillin, 10 mg/mL streptomycin, 1 mg/mL amphotericin B; Gibco), and 2 mM l-glutamine (Gibco).

For the highest test concentration (C8), 100 mg of each sample were weighed and dissolved in a DMSO:D10 mixture (D10 = DMEM + 10% FBS, Vitrocell) to obtain a stock solution; serial 1:2 dilutions yielded eight concentrations from 50 mg/mL (C8) to 0.39 mg/mL (C1). In this preparation, the final solvent content in assay wells did not exceed 0.5% (v/v). Vehicle control assays (solvent under identical dilution conditions, without extract) were performed in parallel and confirmed that the observed effects were not attributable to the solvents.

Assays were performed in 96-well plates (Corning, Cat. 3599) seeded with 2 × 10^4^ HaCaT cells/well. Plates were incubated at 37 °C/5% CO_2_ for 24 h, inspected under an inverted microscope (ZEISS Primovert, 40×, model 415510-1101-000, s/n 3842011163), and then exposed to the extracts for an additional 48 h. DMSO was included as vehicle control.

After incubation, medium was removed and wells were washed with 0.9% saline (Equiplex). Then 100 μL of 0.05% (w/v) MTT [3-(4,5-dimethylthiazol-2-yl)-2,5-diphenyltetrazolium bromide, Invitrogen] were added per well and plates incubated for 2 h. Wells were washed again with saline, followed by 100 μL of cold isopropanol (99.5%; Synth) to solubilize the formazan.

In parallel, 10 μL of PrestoBlue were added to a subset of wells, with incubation for 3 h protected from light, followed by gentle shaking for 15 min. Readings were acquired on a BioTek Synergy HT microplate reader (Gen5 software) at 630 nm for MTT and 560/590 nm (excitation/emission) for PrestoBlue. Cell viability was calculated from the absorbance/fluorescence values of treated wells relative to the mean of cell controls (set as 100%). Background signal was corrected using wells containing culture medium plus the corresponding reagent (no cells) as blanks.

A final DMSO concentration of 0.5% (v/v) was present in all wells during treatment with the extracts. To assess any potential effect of DMSO itself on cell viability, DMSO was also tested separately over a concentration range of 0.039–5 μL/mL.

IC_50_ and IC_90_ values were calculated by linear interpolation from the concentration-response data, corresponding to the concentrations that reduced cell viability by 50% and 90%, respectively, relative to the mean of cell controls (set as 100%). Experiments were performed in triplicate, and results are reported as mean ± standard deviation. To estimate the approximate LD_50_ of the extracts, IC_50_ values obtained from the in vitro basal cytotoxicity assay were converted using the regression model described in OECD Guideline No. 129 (2010) for substances with no defined molecular weight eq 1.^23^

1 $${\mathrm{log LD}}_{50}\left(\mathrm{mg}/\mathrm{kg}\right)=0.372\times {\mathrm{log IC}}_{50}\left(\mu g/\mathrm{mL}\right)+2.024$$

Extracts were classified according to the Globally Harmonized System of Classification and Labeling of Chemicals (GHS). This approach has inherent limitations, as in vitro cell culture systems do not fully replicate whole animal physiology in terms of distribution, metabolism, and toxicokinetics. Therefore, the resulting values are approximate indicators of acute toxicity.

#### Cytotoxic Activity in Spheroid Model

In addition to the monolayer assays, crude extracts AHB and AHK were evaluated for cytotoxicity in three-dimensional (3D) HaCaT spheroid cultures. Experiments followed the same culture and incubation conditions described above, in line with OECD Guideline No. 129 (2010),^23^ using 37 °C, 5% CO_2_, and 97% relative humidity.

To initiate spheroid formation, 10 μL of D10 medium were added to the peripheral wells of 96-well plates to minimize evaporation. The central 60 wells received 100 μL of HaCaT cell suspension in D10. For cell magnetization, NanoShuttle (Kit Bio-Assembler, n3D Bioscience, Inc.) was added to the suspension, which was then centrifuged until a brownish pellet was observed. Cells were plated and the plate was placed on a magnetic drive for 24 h to induce spheroid assembly, followed by an additional 24 h in the incubator.

Extract solutions were prepared in DMSO:D10 with vortex mixing (Corning LSE, model 6775, s/n S6020525). 25 mg/mL and 100 mg/mL for AHK, and 40 and 100 mg/mL for AHB. After removal from the incubator, plates were inspected using an inverted microscope (ZEISS Primovert, 40×, model 415510-1101-000, s/n 3842011163). The culture medium was aspirated, and extract solutions, vehicle control (DMSO), and cell controls were added, with each column corresponding to one experimental condition, followed by incubation for 48 h at 37 °C and 5% CO_2_. The negative control (CC−) consisted of untreated cells, while the positive control (CC+) consisted of cells exposed to a high concentration of DMSO. All assays were performed in triplicate. The final concentration of DMSO in the wells was 0.5% (v/v) for solubilization purposes. DMSO was also included as a vehicle control at a single concentration of 20% to assess its effect on spheroid viability.

After incubation, medium was aspirated and wells were washed with 0.9% saline (Equiplex). Then 100 μL of 0.05% (w/v) MTT solution were added per well and plates were incubated for 2 h at 37 °C. The MTT solution was removed, 100 μL of isopropanol (99.5%; Synth) were added to each well, and plates were gently shaken on an orbital shaker (Biosan, 3D type, s/n 010151-1304-0299) for 15 min. Absorbance was read on a BioTek Synergy HT microplate reader (Gen5 software) at 630 nm. Cell viability in 3D spheroid cultures was determined from the absorbance/fluorescence values of treated spheroids relative to the mean of untreated controls (set as 100%). Background absorbance associated with cellular metabolism was corrected using culture medium as the blank. Data were analyzed by one-way ANOVA followed by Tukey’s post hoc test, considering differences statistically significant at *p* < 0.05.

#### Elastase Inhibition Assay

Elastase inhibition by the extracts and its hexane, ethyl acetate and hydromethanolic fractions was assessed using porcine pancreatic elastase (E.C.3.4.21.36; Sigma-Aldrich, St. Louis, MO, USA) and the substrate *N*-succinyl-Ala-Ala-Ala-*p*-nitroanilide (Suc-Ala3-*p*NA, S4760, Sigma-Aldrich), following established procedures.^14,24,25^ Samples were dissolved in DMSO to prepare a stock solution of 31 mg/mL, which was then diluted in a 50% DMSO/ultrapure water mixture to obtain a solution of 3,100 μg/mL. The positive control, quercetin, was tested at the same concentration of 100 μg/mL as the samples. The analyses were performed in duplicate, and the results were subjected to one-way ANOVA followed by Tukey’s post hoc test (significance set at *p* < 0.05).

Reactions were conducted in a final volume of 725 μL containing 87 mM Tris-HCl buffer (pH 8.0), 0.2 μg/mL elastase, and 100 μg/mL sample. Mixtures were incubated at 25 ± 1 °C for 15 min, after which 0.3 mM Suc-Ala3-*p*NA was added to initiate the reaction. Four controls were included: blank (Trizma buffer +1.6% DMSO), negative control (Trizma buffer +1.6% DMSO + enzyme), sample control (Trizma buffer + sample only), and positive control (Trizma buffer + enzyme + DMSO + quercetin). The final DMSO concentration in the assay was approximately 1.6% v/v for all samples and controls. Elastase activity was monitored at 410 nm for 5 min using a 1 cm path-length cuvette on a Cary 60 UV–vis spectrophotometer (model G6860A; s/n MY24239213; Agilent Technologies, Santa Clara, CA, USA). Sample and solvent blanks were included and used as references to account for any background absorbance.

Inhibition (%) was calculated using eq 2, where Ai and Af are the initial and final absorbance values of the sample, and Aic and Afc are the initial and final absorbance values of the negative control (enzyme-substrate).

2 $$\mathrm{Inhibition}\%=100-100\times \left(\frac{\mathrm{Af}-\mathrm{Ai}}{\mathrm{Afc}-\mathrm{Aic}}\right)$$

#### Tyrosinase Inhibition Assay

Samples (extracts and fractions) were evaluated against mushroom tyrosinase (EC 1.14.18.1; Y3824, Sigma-Aldrich) using l-3,4-dihydroxyphenylalanine (l-DOPA) (D9628, Sigma-Aldrich) as substrate, with spectrophotometric monitoring at 475 nm after 5 min at 25 ± 1 °C.^24,26,27^ The enzyme solution, containing 30 U/mL tyrosinase in 20 mM phosphate buffer (pH 6.5), was preincubated with the test sample or kojic acid (positive control) at a final concentration of 100 μg/mL. The reaction mixture (final volume 709 μL) was prepared by combining phosphate buffer, ultrapure water, the sample, and enzyme solution, and incubated for 15 min at 25 ± 1 °C prior to substrate addition to initiate the reaction.

Assays were performed in duplicate with the following controls: blank (phosphate buffer + 1.6% DMSO), negative control (phosphate buffer + 1.6% DMSO + tyrosinase), sample control (phosphate buffer + sample), and positive control (phosphate buffer + kojic acid + tyrosinase). To account for possible absorbance interference, sample and solvent blanks lacking either enzyme or substrate were also recorded and subtracted from the readings. Immediately after adding l-DOPA (final concentration of 0.5 mM), absorbance at 475 nm was recorded using a 1 cm path-length cuvette on a Cary 60 UV–vis spectrophotometer. Tyrosinase inhibition was calculated as in eq 2. Results were analyzed using one-way ANOVA followed by Tukey’s post hoc test, and differences were considered significant at *p* < 0.05.

## Results and Discussion

### ^1^H NMR

^1^H NMR spectroscopy was used to investigate the chemical profiles of the crude extracts and their corresponding fractions from AHB and AHK. As these samples consist of complex mixtures, the ^1^H NMR data were interpreted at the level of predominant spectral features and compound classes and were not used for unequivocal identification of individual metabolites.

The crude extracts showed highly similar spectra with substantial signal overlap (Figure S1). The signals observed are consistent with low-polarity to nonpolar constituents, including terpenes and long-chain acids (δ 0.7–1.7 and δ 5.0–5.5), such as fatty acids, cardols, cardanols, and anacardic acids.^11^ In addition, signals attributable to carbinolic/oxymethine protons support the presence of free saccharides as well as saccharides associated with other metabolites and methoxy substituents.

The hexane fractions (AHBHex and AHKHex) displayed comparable ^1^H NMR features (Figure S2), with profiles dominated by resonances consistent with nonpolar lipid-like constituents, such as neutral lipids/triacylglycerols and long-chain aliphatic chains. Signals observed in the δ 4.0–4.33 region are consistent with protons from glycerol backbone protons, while resonances in the δ 5.23–5.46 range are consistent with olefinic protons from unsaturated fatty acyl chains.^28^ Given the complexity of these mixtures, no specific lipid species or carotenoids were assigned based solely on ^1^H NMR data.

The ^1^H NMR spectra of the ethyl acetate fractions (AHBAc and AHKAc; Figure S3) showed two doublets at δ 6.57 and δ 7.80 (both *J* = 16.01 Hz), characteristic of a *trans*-alkenyl system. Two additional doublets between δ 6.70 and δ 7.22 are consistent with an AA′BB′ aromatic pattern of a *para*-disubstituted benzene ring.

These coupling patterns and chemical shifts are highly consistent with a major *trans*-hydroxycinnamate component and are compatible with *p*-coumaric acid **(1)** (Figure 1),^29^ as reported in the literature, including studies on peels and pulp of *A. occidentale* pseudofruit.^12^ The chemical shift values and coupling constants observed in the ethyl acetate fractions, together with the corresponding literature data, are summarized in Table 1. Given the mixed nature of these fractions, the assignment is interpreted at the level of a major component based on literature comparison.

**Figure 1:**
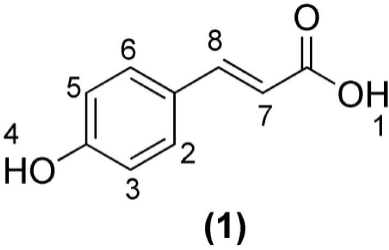
Structure of *p*-coumaric acid **(1)**, shown for comparison with the ^1^H NMR features observed in the ethyl acetate fractions.

**Table 1:** ^1^H NMR Chemical Shifts of the Major *trans*-Hydroxycinnamate Signals Observed in the Ethyl Acetate Fractions (MeOD-*d_4_*, 400 MHz), Compared with Literature Data for *p*-Coumaric Acid **(1)**, Supporting a Literature-Consistent Major-Component Assignment

Hydrogen	Literature^a^ δH (mult, *J* in Hz)	AHBAc δH (mult, *J* in Hz)	AHKAc δH (mult, *J* in Hz)
2, 6	7.53 (m)	7.23 (d, *J* = 8.16)	7.22 (d, *J* = 8.12)
3, 5	6.88 (m)	6.72 (d, *J* = 8.16)	6.70 (d, *J* = 8.12)
7	6.32 (d, *J* = 15.95)	6.57 (d, *J* = 16.01)	6.58 (d, *J* = 16.01)
8	7.59 (d, *J* = 15.95)	7.80 (d, *J* = 16.01)	7.80 (d, *J* = 16.01)

a Spectrum recorded at 300 MHz in deuterated acetone.^30^

*p*-Coumaric acid, the most abundant hydroxycinnamic acid isomer, occurs in plants in free and conjugated forms and serves as a precursor to diverse phenolics. Multiple biological activities have been reported, including antioxidant, anti-inflammatory, antimutagenic, antiulcer, antitumor, antidiabetic, cardioprotective, neuroprotective, analgesic, and anxiolytic effects,^31^ supporting the dietary relevance of *p*-coumarate-containing foods. In cell-based assays, *p*-coumaric acid displayed marked antioxidant effects in endothelial and lens epithelial cells and in keratinocytes exposed to UV radiation; *in vivo*, it reduced oxidative stress beyond vitamin E and decreased DNA damage in guinea-pig colonic mucosa.^32,33^

For the hydromethanolic fractions (AHBAq and AHKAq; Figure S4), the ^1^H NMR spectra showed low-intensity signals at δ 6.0–8.5 indicative of additional aromatic compounds, as well as resonances below δ 2.0 consistent with aliphatic chains. In the carbinolic region (δ 3.0–4.0), signals suggest methoxy substituents and the presence of saccharides and/or glycosylated metabolites. The occurrence of glycosylated constituents is well documented in *Anacardium* species.^12^

Overall, the compound classes inferred from the NMR data mirror the chemical complexity of the samples and align with activities of interest—antioxidant, anti-inflammatory, and antimicrobial^34^—that are relevant to cosmetic applications.

### Molecular Network Analysis

The molecular networks comprised 106 ions in positive ionization mode and 46 ions in negative mode, with a total of nine compounds putatively annotated through the GNPS platform. Metabolite annotations were reported as putative (MSI level 2) and were not confirmed with authentic standards. Annotations were supported by LC-MS chromatograms (Figures S5–S8), MS/MS spectra (Figures S9–S17), and proposed fragmentation pathways (Figures S18–S26). Molecular network analyses are presented in Figures S27–S30, while the corresponding cosine scores and diagnostic fragment ions for each annotated compound are summarized in Table 2.

**Table 2:** Metabolites Revealed in the Ethanolic Extracts of AHB and AHK, Annotated via GNPS Using HPLC-ESI-QTOF-MS/MS Data Acquired in Both Positive and Negative Ionization Modes

Structure	Metabolite^a^	Cosine (GNPS)	RT (min)	Adduct	*m*/*z* observed	Error (ppm)	MS^2^ fragment ions (*m*/*z*)	Molecular formula	Reference
**2**	disaccharide^a,b^	0.77	0.8	[M + Na]^+^	365.1058	1.09	185.0415, 203.0524, 365.1058	C_12_H_22_O_11_	^35,36^
**3**	peonidin *O*-hexoside^a,b^	0.79	4.4	[M]^+^	463.1226	–1.94	258.0523, 286.0468, 301.0702	C_22_H_23_O_11_^+^	^38,39^
**4**	quercetin *O*-hexoside^b^	0.88	5.1	[M + H]^+^	465.1015	–2.80	153.0183, 257.0438, 285.0378, 303.0484	C_21_H_20_O_12_	^40^
**5**	ginkgolic acid (15:1)^a,b^	0.88	16.8	[M – H]^−^	345.2434	–0.29	119.0421, 133.0652, 175.1122, 301.2531	C_22_H_34_O_3_	^41^
**6**	quercetin *O*-pentoside^b^	0.84	5.6	[M – H]^−^	433.0774	–0.46	151.0033, 178.9972, 227.0702, 255.0291, 271.0243, 300.0265	C_20_H_18_O_11_	^42,43^
**7**	myricetin *O*-pentoside^b^	0.92	5.1	[M – H]^−^	449.0720	–1.11	151.0039, 178.9974, 243.0270, 271.0236, 287.0187, 316.0223	C_20_H_18_O_12_	^42,44^
**8**	quercetin *O*-hexoside^a,b^	0.89	5.3	[M – H]^−^	463.0872	–2.25	151.0027, 178.9971, 227.0352, 255.0297, 271.0241, 300.0266	C_21_H_20_O_12_	^42,43^
**9**	quercetin *O*-hexosyl-deoxyhexoside^a,b^	0.92	5.1	[M – H]^−^	609.1453	–1.31	151.0037, 178.9984, 255.0284, 271.0241, 300.0265	C_27_H_30_O_16_	^42,43^
**10**	qyricetin *O*-hexosyl-deoxyhexoside^a,b^	0.87	4.8	[M – H]^−^	625.1389	–3.36	151.0030, 178.9969, 271.0234, 287.0182, 316.0208	C_27_H_30_O_17_	^42,44^

a Annotations were accepted for cosine scores above 0.6, with a minimum of four matched fragments and a mass tolerance of 0.02 Da. Fragmentation proposals were used to support structure assignment, corresponding to MSI level 2. The presence of compounds in AHB and AHK samples is indicated by a and b, respectively.

The secondary metabolites dereplicated from crude extracts AHK and AHB belonged mostly to the flavonoid class, plus a disaccharide and an anacardic acid. The annotated compounds consisted of a disaccharide **(2)**, peonidin *O*-hexoside **(3)**, quercetin *O*-hexoside **(4)**, ginkgolic acid **(5)**, quercetin *O*-pentoside **(6)**, myricetin *O*-pentoside **(7)**, quercetin *O*-hexoside **(8)**, quercetin *O*-hexosyl-deoxyhexoside **(9)**, and myricetin *O*-hexosyl-deoxyhexoside **(10)**. The analysis revealed that most of the annotated compounds were present in both crude extracts. However, three compounds were exclusively annotated in the AHK extract, namely compounds **4**, **6**, and **7**.

Compound **2** was assigned as a disaccharide based on the ion at *m*/*z* 365.1058, corresponding to the sodium adduct [M + Na]^+^ (neutral mass ≈ 342 Da). The MS/MS spectrum exhibited fragment ions at *m*/*z* 203.0524 (sodiated hexose), which arouse from a neutral loss of 162 Da (hexose unit), and *m*/*z* 185.0415 (subsequent water loss).^35^ These fragments are characteristic of disaccharides composed of two hexose units.^36^ Considering that sucrose, glucose, and fructose are the primary sugars reported in the pseudofruit of *A. occidentale*, compound **2** was annotated as a hexose-based disaccharide, most likely sucrose.^37^

Compound **3** was annotated as peonidin *O*-hexoside, a glycosylated anthocyanin, based on its charged molecule ([M]^+^) at *m*/*z* 463.1226 and characteristic fragments. MS/MS fragmentation is particularly informative for glycosylated flavonoids/anthocyanins because glycosides often produce diagnostic sugar-related product ions (oxonium ions) and/or characteristic neutral losses, which support inference of the sugar units and the aglycone (or flavylium core) in dereplication workflows. For example, sugar-derived fragment ions have been used as evidence for the presence of specific sugars in mass spectrometry-based analyses of natural glycosides.^45^ In this context, the MS/MS fragmentation of compound **3** showed a neutral loss of 162 Da (corresponding to a hexose moiety), yielding the diagnostic aglycone product ion at *m*/*z* 301.0702 (peonidin-type flavylium cation). This transition (*m*/*z* 463 to *m*/*z* 301) is in high agreement with the fragmentation profile reported by Sun et al. (2014) for peonidin 3-*O*-glucoside.^38,39^ Furthermore, the present analysis detected an additional fragment at *m*/*z* 286.0468, which is compatible with the subsequent loss of a methyl group ([M + H – 162 – 15]^+^) from the flavylium core. This further demethylation provides additional structural evidence for the peonidin aglycone.

Compound **5** was annotated as ginkgolic acid (15:1), an anacardic-acid derivative ([M – H]^−^ at *m*/*z* 345.2434). The assignment is supported by its MS/MS pattern, which matches the data reported by Lee et al. (2013).^41^ Specifically, the observed ion at *m*/*z* 301.2531 corresponds to the loss of a neutral CO_2_ molecule (44 Da) from the precursor ion. This compound is primarily found in cashew nut shells,^46,47^ but this and other anacardic acids have also been detected in cashew apple juice.^48^ Likewise, Santos et al. (2023) annotated ginkgolic acid in negative mode (*m*/*z* 345.244) and a structurally related compound, 6-pentadecylsalicylic acid, in positive mode (*m*/*z* 349.183), further supporting the occurrence of these anacardic acid derivatives in *Anacardium* pseudofruits.^10^

Compounds **4**, **6**, **8**, and **9** share the same aglycone, as glycosylated derivatives of quercetin. Compound **4**, annotated in positive mode, undergoes neutral loss of a hexose (162 Da) to *m*/*z* 303.0484, followed by dehydration to *m*/*z* 285.0378 and CO loss to *m*/*z* 257.0438.^40^

Compounds **6** and **8**, observed in negative mode ([M – H]^−^ at *m*/*z* 433.0774 (**6**)/463.0872­(**8**)), exhibited similar fragmentation patterns. In both cases, the MS/MS spectra were characterized by a strong peak referent to the aglycone radical anion at *m*/*z* 300.0265 (**6**)/300.0266 (**8**) resulting from the neutral loss of a pentose (132 Da) and a hexose (162 Da) moiety, respectively. This ion is formed by the homolytic cleavage of the *O*-glycosidic bond, a diagnostic marker for glycosylation at the C-3 position.^49^ Subsequent fragmentation of this quercetin core yielded ions at *m*/*z* 271.0243 (**6**)/271.0241 (**8**) (CO loss) *m*/*z* 255.0291 **(6)**/255.0297 **(8)** (loss of atomic oxygen). Additionally, *m*/*z* 178.9972 **(6)**/178.9971 **(8)** and *m*/*z* 151.0033 **(6)**/151.0027 **(8)** arose from retro Diels–Alder (RDA) cleavage of the flavonoid C-ring at positions 1,2 and 1,3, respectively, further confirming the quercetin skeleton.^42,43^ Compound **9**, also in negative mode, displayed a comparable pattern to **6** and **8**, with additional sugar loss, consistent with a diglycosylated quercetin.

Compounds **7** and **10** were annotated as myricetin glycosides. In both cases, the fragment at *m*/*z* 316.0223 **(7)**/316.0208 **(10)** corresponds to the radical anion of myricetin formed via homolytic cleavage of the *O*-glycosidic bond. Subsequent fragments included *m*/*z* 287.0187 **(7)**/287.0182 **(10)** (CO and H loss) and *m*/*z* 271.0236 **(7)**/271.0234 **(10)** (loss of atomic oxygen). Diagnostic ions at *m*/*z* 178.9974 **(7)**/178.9984 **(10)** and *m*/*z* 151.0039 **(7)**/151.0037 **(10)** from RDA cleavage at 1,2 and 1,3 further supported these assignments.^44^

The compounds revealed by molecular networking corroborate the NMR observations, including signals observed in the aromatic region (δ 6.0–8.5) and carbinolic/oxymethine region (δ 3.0–4.0). Notably, the disaccharide **(2)** was annotated exclusively based on MS/MS fragmentation patterns obtained through GNPS molecular networking (see Figure S18) and from the ^1^H NMR spectra. Compounds structurally related to quercetin *O*-pentoside **(6)**, quercetin *O*-hexoside **(8)**, and quercetin *O*-hexosyl-deoxyhexoside **(9)** have already been reported in the pseudofruit of *A. occidentale* - identified as guaijaverin, hyperoside, and rutin—as well as aglycone myricetin.^50–53^ Additionally, the chemical profile observed here resembles that reported by Santos et al. (2023) for hydromethanolic extracts of *A. humile* collected in Goiás.^10^ These findings highlight the potential of these species as sources of bioactive compounds with possible applications in the cosmetic sector, particularly in the modulation of skin-related enzymes such as elastase and tyrosinase. The structures are summarized in the Venn diagram in Figure 2.

**Figure 2:**
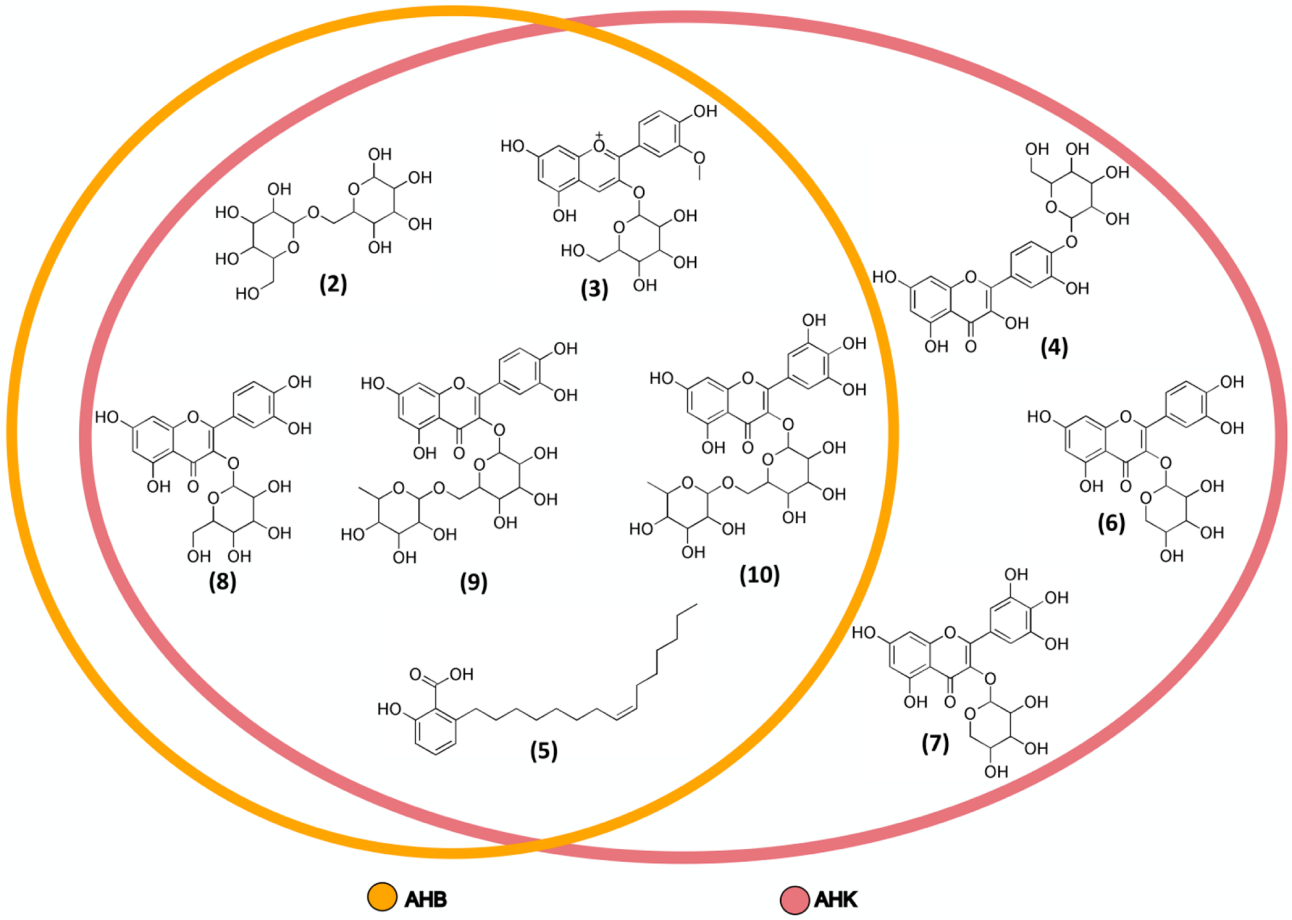
Venn diagram illustrating the distribution of compounds annotated in AHB (yellow) and AHK (red) ethanolic extracts, highlighting overlap and exclusivity across samples.

### Thermal Stability

Thermal stability defines the temperature range over which a material can be heated without undergoing decomposition that compromises its chemical integrity and functionality. This parameter is essential for evaluating the compatibility of active compounds with excipients in industrial formulations.^54^

The analyses were performed over a temperature range of 30–800 °C, under an simulated oxidizing atmosphere. The TG-DTG curves for AHB and AHK are shown in Figure S31, with the corresponding thermal data summarized in Table S1.

Both samples exhibited initial mass losses between 30 °C (T_onset) and 120 °C, corresponding to the release of residual moisture and volatile compounds.^55^ The maximum rate of mass loss (T_max) within this range was observed at 91.82 °C for AHB and 91.61 °C for AHK, with overall mass losses of 13.46% and 12.57%, respectively, indicating low-molecular-weight constituents in both extracts.

The thermal events from just above 120 to 270 °C were marked by the most significant mass losses in both samples, with decomposition peaking at T_max = 205.53 °C for AHB and T_max = 205.84 °C for AHK, corresponding to mass losses of 41.60% and 42.35%, respectively. These losses are attributed to the thermal decomposition of phenolic compounds (e.g., flavonoids),^56^ and carbohydrates.^57^ This finding is consistent with the chemical profiles obtained by ^1^H NMR analysis of AHK and AHB, which indicated a notable content of carbohydrates.

The DTG curves of both extracts showed progressive mass loss without a return to baseline between thermal events, indicating overlapping decomposition steps. Complete thermal degradation was observed above 570 °C, with final residue reaching 0%, corresponding to 571.70 °C for AHB and 579.59 °C for AHK, suggesting a low inorganic content in both samples.

Based on the observed thermal behavior, both samples appear suitable for processes involving heating up to approximately 120 °C, as mass loss in this range is primarily attributable to volatilization of residual solvent and low-molecular-weight metabolites rather than decomposition of thermally stable constituents.

### Photochemical Study

The ethanolic extracts of AHB and AHK exhibited thermochromism, shifting from yellow at low temperatures to orange for AHB and red for AHK at room temperature and above. Ground-state photophysical characterization of the extracts was conducted in a methanol:water (1:3, v/v) solution at −10 and 24 °C (Figure S32).

At both temperatures, the extracts exhibited qualitatively similar absorption profiles, with maximum absorption centered at approximately 460 nm for AHB and 520 nm for AHK at 24 °C. At −10 °C, the overall spectral shape was preserved, but the visible bands showed markedly reduced intensity, preventing accurate determination of the absorption maxima in this region. This behavior suggests that temperature variation mainly affects the extent of chromophore association or aggregation, rather than causing a substantial shift in electronic transitions. Accordingly, the intense colors observed in the ethanolic extracts at room temperature likely arise from intermolecular interactions in the condensed phase, which are largely disrupted in solution and govern the thermochromic response.

The thermochromic properties of both extracts are plausibly attributed to interactions between flavonoids (including anthocyanidins) and fatty acids as well as ginkgolic (anacardic) acids. At temperatures below 0 °C, the extracts exhibit a more solid-like appearance that transitions upon heating. As the temperature increases, the extracts undergo a phase change to a more liquid state, enabling acid–base and π–π/hydrogen-bonding interactions between the fatty acids and anthocyanidins, which in turn can result in a visible color shift.^58–60^

Additionally, the extracts present absorption maxima in the UV region, mainly below 300 nm, which corroborates the presence of UV-active compounds, particularly phenolics/flavonoids. These compounds play key physiological roles in plants, including protection against UV-induced damage.^61^ Although the maxima are observed in the lower-UV range, both extracts showed appreciable absorbance across the broader UV spectrum, consistent with the presence of multiple aromatic and conjugated structures.

The effects of exposing the extracts to ultraviolet radiation at 254 and 363 nm, simultaneously, were also evaluated. The UV–vis absorption spectra of the extracts before and after UV exposure (Figure S33) exhibit similar profiles, and structural modifications in the chromophore groups present in the extracts occurred to a limited extent.

The absorption maxima and optical behavior observed in the extracts are compatible with properties desirable for natural sunscreen agents/colorants, which could serve as alternatives to synthetic compounds and add value to Cerrado-derived products. Moreover, these extracts exhibit characteristics suitable for use as natural colorants in food, representing a potentially healthier and more sustainable substitute for artificial dyes. Nevertheless, additional research is necessary to define application limits and address safety aspects.

### Cytotoxic Activity

Cytotoxicity assays are widely applied to assess the safety profile of substances used in agrochemical, pharmaceutical, food, and cosmetic formulations. These tests provide information on parameters such as cell viability, proliferation, membrane integrity, and metabolic activity following exposure to a given compound.^62^ In the present study, the HaCaT cell line was used in combination with the vital dyes MTT and PrestoBlue. Table 3 shows the results obtained for the ethanolic extracts (AHB and AHK).

**Table 3:** IC_50_, IC_90_, and LD_50_ Values of the Extracts Evaluated in This Study on Human Keratinocytes (HaCaT Cell Line)^a^

Dye	Extract	IC_50_ (mg/mL)	IC_90_ (mg/mL)	LD_50_ (mg/kg)	Hazard level
MTT	AHB	41.330 ± 1.618	28.539 ± 0.609	5511.25	5 (safe for skin contact)
	AHK	0.891 ± 0.079	0.708 ± 0.259	1322.37	4 (caution)
PrestoBlue	AHK	21.281 ± 0.786	17.849 ± 0.604	4305.39	5 (safe for skin contact)

a **Legend:** values are mean ± standard deviation of three independent experiments. Hazard levels follow the globally harmonized system of classification and labeling of chemicals (GHS). AHB: ethanolic extract of pseudofruits from Brazlândia; AHK: ethanolic extract of pseudofruits from Kalunga community. DMSO entries refer to the final vehicle concentration in wells.

The extracts AHB and AHK exhibited distinct cytotoxicity profiles in the MTT assay. AHK showed significantly higher toxicity in HaCaT cells, with an IC_50_ value of 0.891 ± 0.079 mg/mL, while AHB presented an IC_50_ of 41.330 ± 1.618 mg/mL, indicating that AHK is ∼40-fold more cytotoxic under these conditions. Based on the estimated LD_50_ values from the MTT assay, AHK was classified as hazard level 4, indicating a need for caution in its use on skin. In contrast, AHB was classified as hazard level 5 (LD_50_ = 5511.25 mg/kg), representing a lower risk of toxicity upon skin contact. When tested using PrestoBlue, only AHK was evaluated, showing an LD_50_ of 4305.39 mg/kg, which corresponds to hazard level 5 and indicates lower apparent toxicity than suggested by the MTT assay.

Background absorbance due to cellular metabolism was corrected using culture medium as the blank. The final DMSO concentration in the wells was 0.5% (v/v). The observed discrepancy is likely caused by the extract’s pigmentation affecting the MTT assay. In addition, the solvent DMSO was tested as a control and did not reduce cell viability below 50% in either assay, which prevented the determination of IC_50_ values; however, in the MTT assay it decreased viability to levels below 90%, allowing the calculation of the IC_90_ (4.827 ± 2.301 μL/mL). These findings confirm that the cytotoxic effects observed are attributable to the extracts rather than solvent interference.

A study conducted by Cefali et al. (2020) evaluated the safety of *A. occidentale* pseudofruit extract in keratinocyte cultures at concentrations ranging from 15 to 250 μg/mL, which are lower than those tested in the present study, and reported no signs of cytotoxicity.^63^ The extract was considered safe for topical use, consistent with the low-to-moderate toxicity observed for both AHB and AHK.

### Cytotoxic Activity in a Spheroid Model

Although the two-dimensional (2D) monolayer culture is the most commonly used model for assessing compound safety, it does not accurately reproduce the complexity of in vivo tissue architecture, which involves multiple interacting cell layers. To address this limitation, three-dimensional (3D) spheroid cultures are used, as they better reproduce the structural and functional features of real tissues.^64^ Figure 3 shows the cell viability data obtained for the spheroid model.

**Figure 3:**
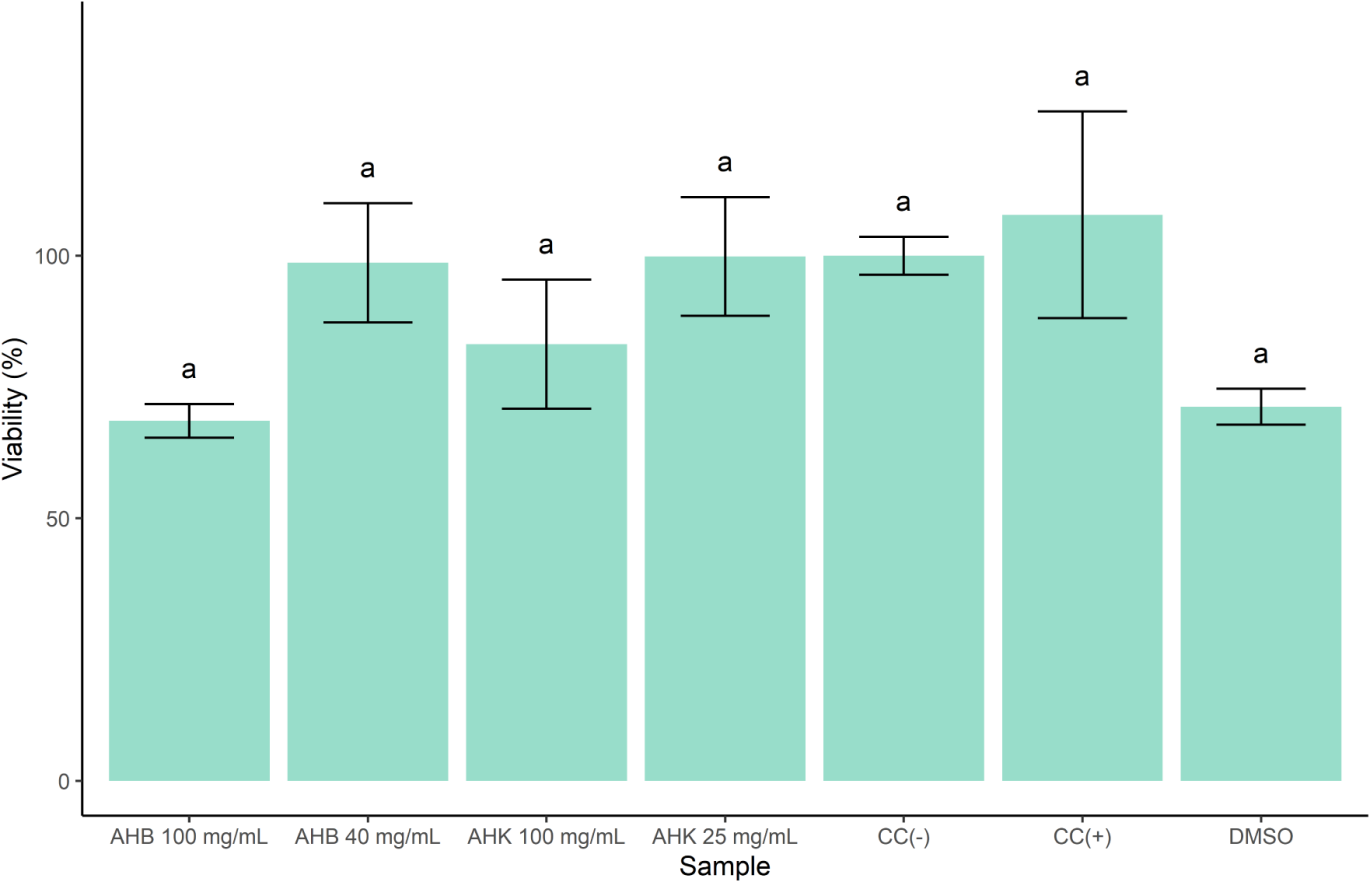
Cell viability of crude AHB and AHK extracts tested at 100 mg/mL and at each sample’s IC_50_. Results are mean ± standard error (SE) (%), *n* = 3. Groups sharing the same letter are not statistically different (one-way ANOVA followed by Tukey’s test, *p* < 0.05). Abbreviations: AHB: crude extract from Brazlândia pseudofruits; AHK: crude extract from Kalunga pseudofruits; CC(−)—negative control; CC­(+)—positive control; DMSO—vehicle.

In this assay, both AHB and AHK exhibited higher tolerance to toxicity compared to the 2D model. Cell viability remained near 100% at concentrations of 40 mg/mL for AHB and 25 mg/mL for AHK, without significant reduction. At 100 mg/mL, a decrease in cell viability was observed, reaching 68.57 ± 3.20% for AHB and 83.19 ± 12.31% for AHK. This behavior is in line with what is expected for the 3D culture model, where the spatial organization of cells enables extensive cell–cell interactions and the formation of diffusion gradients of oxygen, nutrients, and xenobiotics.^65^ These features act as protective barriers, limiting the uniform penetration and activity of test substances throughout the spheroid.^64^

Sousa et al. (2021) reported that the lyophilized extract of *A. occidentale* pseudofruit exhibited no toxicity in Zebrafish (*Danio rerio*), with an LD_50_ above 200 mg/kg.^66^ These findings suggest that the combined extracts of *A. occidentale* and *A. humile* pseudofruits present low toxicity toward human keratinocytes and are likely safe for use in products intended for skin contact.

### Enzyme Inhibitory Activity

Elastase and tyrosinase are enzymes with recognized relevance to the skin aging process. Elastase participates in the degradation of extracellular matrix proteins, and its overexpression has been associated with inflammatory conditions and the acceleration of skin aging, primarily due to collagen and elastin breakdown.^67^ Tyrosinase plays a central role in melanin biosynthesis, and increased activity of this enzyme has been linked to disorders such as hyperpigmentation and spot formation.^14^ Natural inhibitors of these enzymes can be employed in the development of cosmetic formulations aimed at improving skin elasticity and lightening dark spots.

The crude ethanolic extracts (AHB and AHK) and their respective fractions were evaluated for elastase and tyrosinase inhibitory activities, as illustrated in Figure 4. Quercetin and kojic acid were used as positive controls for elastase and tyrosinase, showing inhibition values of 100.00 ± 0.00% and 92.91 ± 2.55%, respectively, at the concentration of 100 μg/mL.

**Figure 4:**
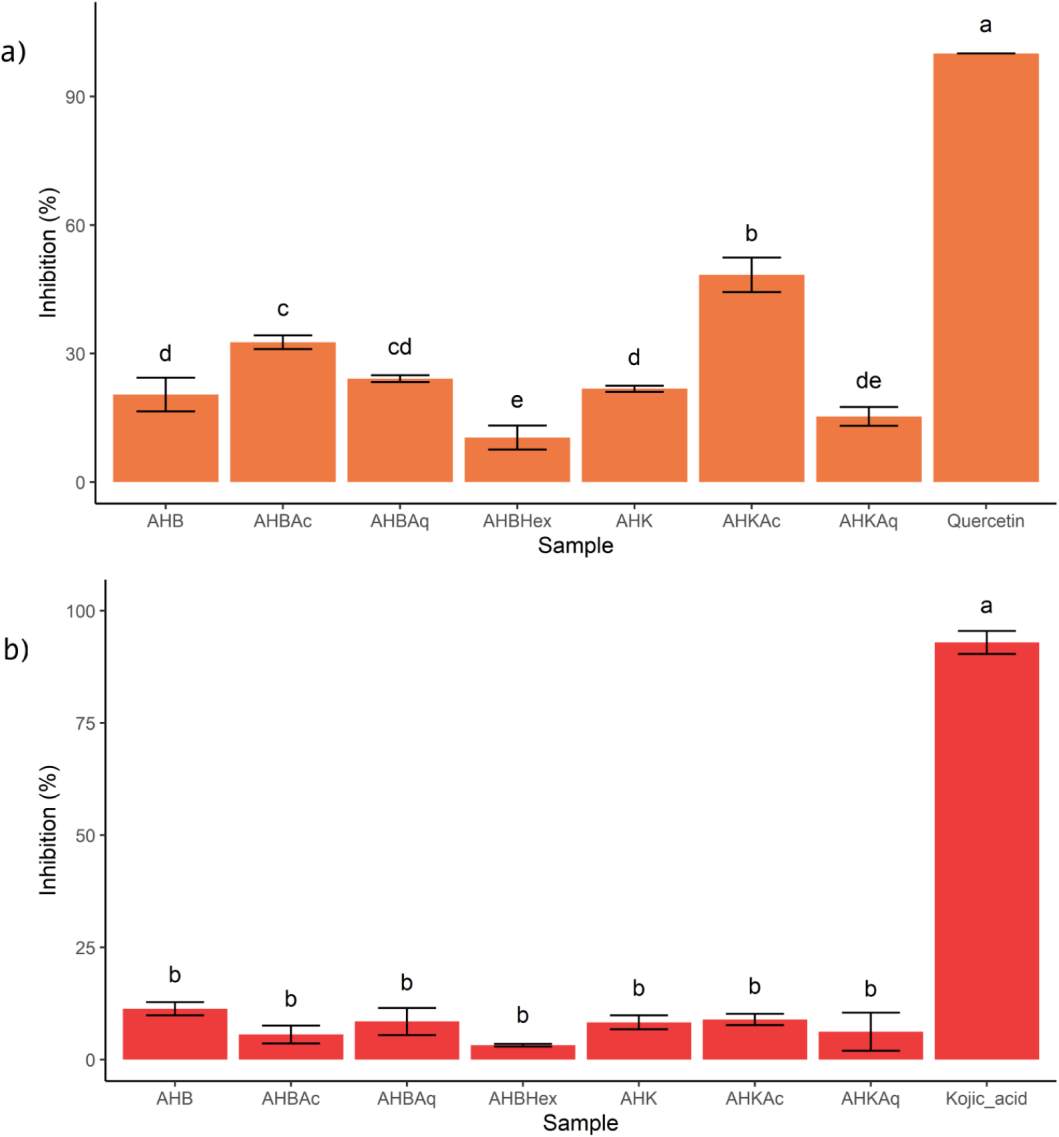
Inhibitory effects on (a) elastase and (b) tyrosinase of crude ethanolic extracts and fractions from AHB and AHK at 100 μg/mL. Results are presented as mean ± standard deviation (SD). Groups sharing the same letter are not significantly different (one-way ANOVA followed by Tukey’s test, *p* < 0.05). Abbreviations: AHB—ethanolic extract from Brazlândia pseudofruits; AHK—ethanolic extract from Kalunga pseudofruits; AHBHex—hexane fraction of AHB; AHBAc—ethyl acetate fraction of AHB; AHBAq—hydromethanolic fraction of AHB; AHKAc—ethyl acetate fraction of AHK; AHKAq—hydromethanolic fraction of AHK.

The crude extracts AHB and AHK exhibited elastase inhibition of 20.42 ± 3.94% and 21.80 ± 0.70%, respectively, which is within the range of relatively moderate activity reported for some plant extracts under similar assay conditions.^68^ The comparable levels of enzyme inhibition are consistent with the chemical annotation results, which indicated a strong similarity in their compound profiles. In contrast, the ethyl acetate fractions AHBAc and AHKAc exhibited higher inhibition levels of 32.65 ± 1.58% and 48.38 ± 4.01%, respectively. The hydromethanolic fraction AHBAq also showed greater inhibition than its corresponding crude extract, with 24.14 ± 0.80%. However, Tukey’s test revealed no statistically significant difference between the inhibition produced by AHB and AHBAq. The results suggest that the compounds responsible for the observed biological activity exhibit intermediate to high polarity, as the hydromethanolic fraction of AHB and the ethyl acetate fraction of AHK showed higher inhibition percentages than their respective crude extracts.

According to Chaikhong et al. (2023), *A. occidentale* leaf extract inhibited elastase by 84.78 ± 2.16% at 500 μg/mL, but showed no activity at 100 μg/mL.^69^ In contrast, the ethyl acetate fractions of AHB and AHK reached inhibition levels of up to 48.38 ± 4.01% at 100 μg/mL, demonstrating that fractionation enhanced activity at this concentration. Although classified as moderate inhibitors,^67^ both AHBAc and AHKAc displayed statistically significant effects. Given that annotation revealed compounds with reported antielastase properties, these fractions may represent promising candidates for cosmetic formulations with antiaging potential.

The aglycones of the annotated flavonoids have been reported to exhibit inhibitory activity against elastase.^70,71^ However, in the present study, elastase inhibition by the crude extracts AHB and AHK, as well as their respective fractions obtained by liquid–liquid partitioning, remained below 50%. Although these flavonoids were annotated, the observed inhibition values suggest that structural variations and glycosylation patterns may influence their biological activity.

Jakimiuk et al. (2021), in a review of the elastase inhibitory activity of flavonoids, emphasized the importance of the catechol group (hydroxyls at positions 3′ and 4′ of the B-ring) for effective inhibition. Methylation at one of these positions generally reduces activity, while certain *O*-methylations elsewhere may enhance it.^72^ Furthermore, the presence of an additional hydroxyl substituent in the B-ring, as in myricetin (3′, 4′, and 5′ hydroxyls), decreases elastase inhibitory activity. Certain substitutions, such as the 3-*O*-rhamnosyl group, were also found to reduce the inhibitory effect of quercetin. These results suggest that, despite the presence of flavonoids with known elastase inhibitory activity, the moderate inhibition observed in the crude extracts and fractions may be due to the low concentration of active compounds within the extracts and their specific structural features.

In addition, the extracts were also tested against tyrosinase, with kojic acid as the positive control. All extracts and fractions exhibited low inhibition values. The AHBAq and AHKAc fractions showed the highest tyrosinase inhibition percentages, with 8.50 ± 3.01% and 8.95 ± 1.24%, respectively. In comparison, the study by Zeitoun et al. (2020) demonstrated that the hydroethanolic extract of *A. occidentale* pseudofruit exhibited tyrosinase inhibitory activity above 10%, but only at concentrations higher than 1000 μg/mL.^73^

Although the tested samples showed low overall tyrosinase inhibitory activity, their chemical profiles suggest the presence of compounds previously reported to possess antityrosinase properties.^16^ In particular, the ^1^H NMR features observed in the AHBAc and AHKAc fractions are compatible with a major *trans*-hydroxycinnamate component, such as *p*-coumaric acid, based on comparison with literature data, while flavonoid *O*-glycosides such as quercetin and myricetin derivatives were predominant in the crude extracts, as revealed by HPLC-ESI-QTOF-MS/MS profiling, linking the observed activity to the chemical composition of the samples. According to An et al. (2010), *p*-coumaric acid is a much weaker inhibitor of mushroom tyrosinase compared to kojic acid but inhibits human tyrosinase approximately 100-fold more effectively.^74^ In addition, *p*-coumaric acid showed a potent antimelanogenic effect in human epidermal cells exposed to UVB. Enriching the crude extracts of *A. occidentale* and *A. humile* pseudofruits with *p*-coumaric acid, either through different extraction methods or further fractionation, may represent a promising strategy to improve their effectiveness against skin hyperpigmentation. Although the tyrosinase tested in this study was from mushroom, and not human, the low response observed here does not invalidate the potential depigmenting activity, particularly considering the known inhibitory effects of *p*-coumaric acid on human tyrosinase.

## Conclusions

The ethanolic extracts of *A. humile* and *A. occidentale* pseudofruits contain flavonoids, carotenoids, and phenolic acids. Cytotoxicity assays demonstrated low toxicity in both monolayer and 3D spheroid HaCaT models, supporting their safety for potential skin-contact applications. Moderate inhibitory activity against elastase and tyrosinase was observed, and compounds such as *p*-coumaric acid, annotated in the extracts, may contribute to these effects. Fractions enriched in *p*-coumaric acid could further enhance antityrosinase activity through optimized extraction or fractionation strategies. Additionally, the UV absorption properties of the extracts highlight their potential use as natural colorants, offering an alternative to synthetic dyes in cosmetic formulations. Overall, these findings support the further evaluation of *A. humile* and *A. occidentale* extracts as sustainable ingredients for cosmetic applications.

## Supplementary Material



## Notes

Single PDF file containing: ^1^H NMR spectra of all crude extracts and fractions; proposed fragmentation mechanisms for the dereplicated compounds with annotated MS/MS pathways; molecular networking data and cluster visualizations; high-resolution MS and MS/MS spectra for all annotated compounds; TG and DTG curves for the crude extracts with a summary table of mass-loss events; and UV–vis absorption spectra from the photochemical study (PDF)

## #

Present Address: Chemistry Institute, Federal University of Rio Grande do Sul, Porto Alegre, RS, Brazil

## Abbreviations

AHB: ethanolic extract obtained from mixed pseudofruits of *Anacardium humile* and *A. occidentale* collected in Brazlândia
AHK: ethanolic extract obtained from mixed pseudofruits of *Anacardium humile* and *A. occidentale* collected in the Kalunga community territory
AHBHex: hexane fraction of the AHB extract
AHBAc: ethyl acetate fraction of the AHB extract
AHBAq: hydromethanolic fraction of the AHB extract
AHKHex: hexane fraction of the AHK extract
AHKAc: ethyl acetate fraction of the AHK extract
AHKAq: hydromethanolic fraction of the AHK extract
^1^H NMR: nuclear magnetic resonance of hidrogen
HPLC-ESI-QTOF-MS/MS: high-performance liquid chromatography coupled to electrospray ionization quadrupole time-of-flight tandem mass spectrometry
ACN: acetonitrile
IC_50_: half maximal inhibitory concentration
IC_90_: concentration required to inhibit 90% of the activity
LD_50_: median lethal dose
