# Supplementary material for: Chemical Profiling and Cosmetic Potential from Anacardium humile and Anacardium occidentale
Source: ACS Omega. 2026 Mar 19;11(12):19220–32. doi: 10.1021/acsomega.5c12447 (PMC13044687; doi:10.1021/acsomega.5c12447)
Supplement: Supplementary file 1 [file ao5c12447_si_001.pdf]

Supplementary Material

CHEMICAL PROFILING AND COSMETIC POTENTIAL FROM *Anacardium humile* AND *Anacardium occidentale*

*Carlos F. da Silva †, Noelle C. F. dos Santos †, Isabella M. do Nascimento †, Cleysla M. da Silva †, Patricia S. Lopes ‡, Larissa S. Costa ‡, Lorena R. F. de Sousa §, Adriana P. M. G. Macedo §, Felipe L. Coelho †, ○, Carlos A. F. Moraes //, Maico R. Severino ⊥, Vanessa G. P. Severino *, †*

†Chemistry Institute, Federal University of Goiás, Esperança Avenue, Goiânia, GO, Brazil.

Corresponding author e-mail: vanessapasqualotto@ufg.br

‡Institute of Environmental, Chemical and Pharmaceutical Sciences, Department of Pharmaceutical Sciences, Federal University of São Paulo, Diadema, SP, Brazil.

§ Chemistry Institute, Federal University of Catalão, Catalão, GO, Brazil.

// Chemistry Institute, Federal University of São Carlos, São Carlos, SP, Brazil.

⊥ Faculty of Sciences and Technology, Federal University of Goiás, Aparecida de Goiânia, GO, Brazil.

^{1}H NMR

Note: The ^{1}H NMR spectra presented here are intended as qualitative chemical fingerprints of the extracts and fractions. Because the samples consist of complex mixtures, the spectra were not used for definitive identification of individual metabolites, but rather to support predominant structural features and compound classes discussed in the main text.

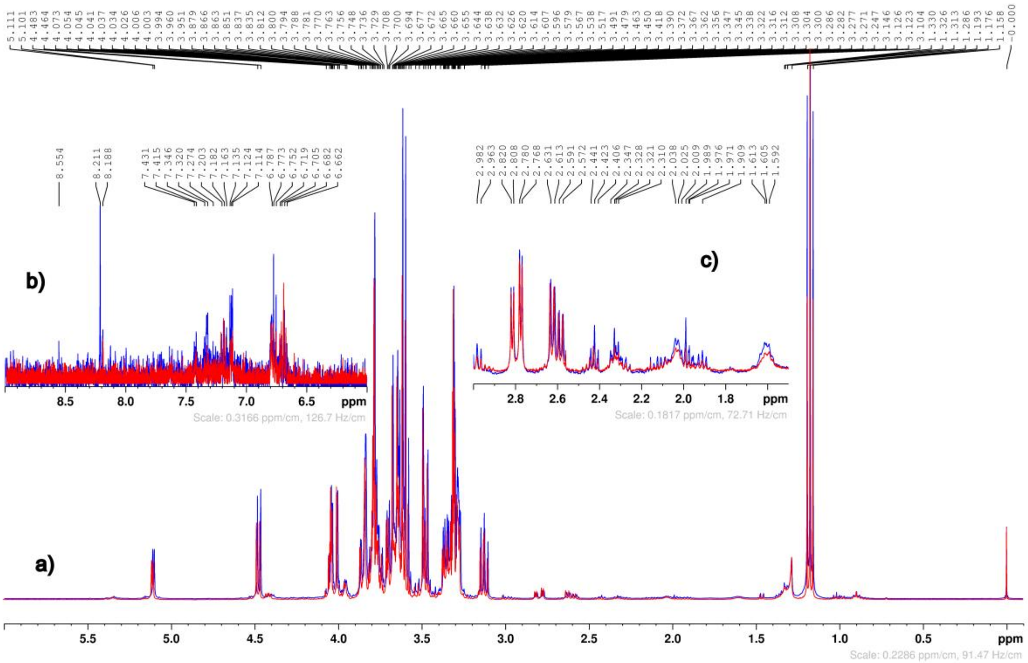

Figure S1. (a) ^{1}H NMR spectra of ethanolic extracts AHB (blue) and AHK (red) recorded in MeOD-d_4. **(b)** Expanded region, δ 6.5–8.5. **(c)** Expanded region, δ 1.5–3.0. Spectra acquired on a 400 MHz spectrometer for proton.

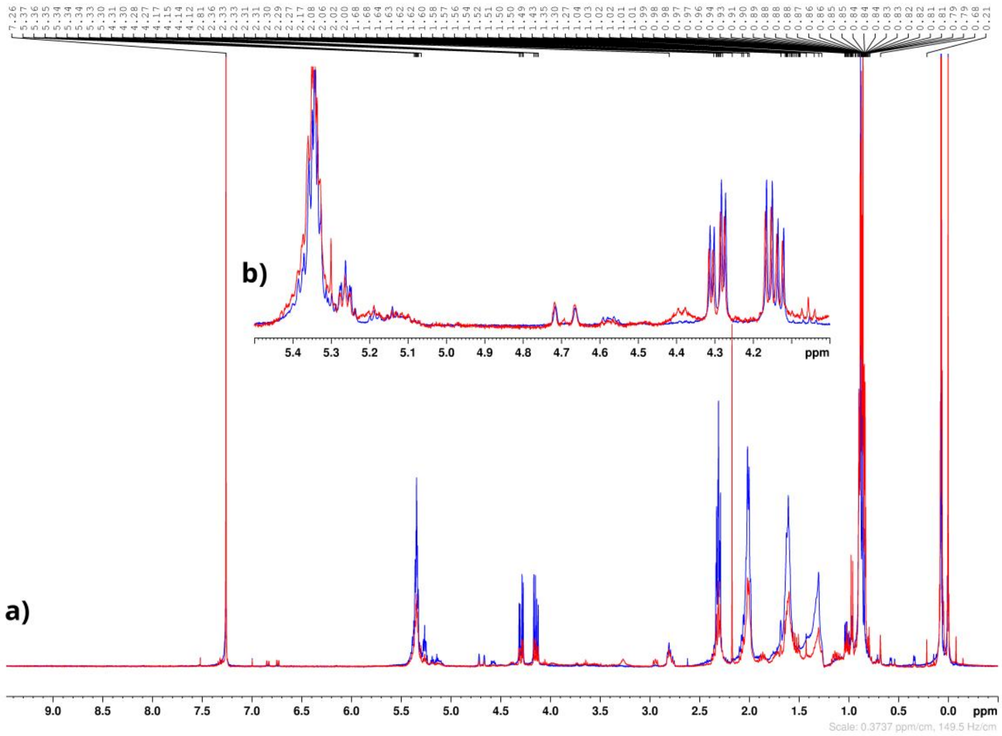

Figure S2. (a) ^{1}H NMR spectra of AHBHex (blue) and AHKHex (red) recorded in $CDCl_3$. **(b)** Expanded region, δ 4.0–5.5, consistent with triacylglycerols protons. Spectra acquired on a 400 MHz spectrometer for proton.

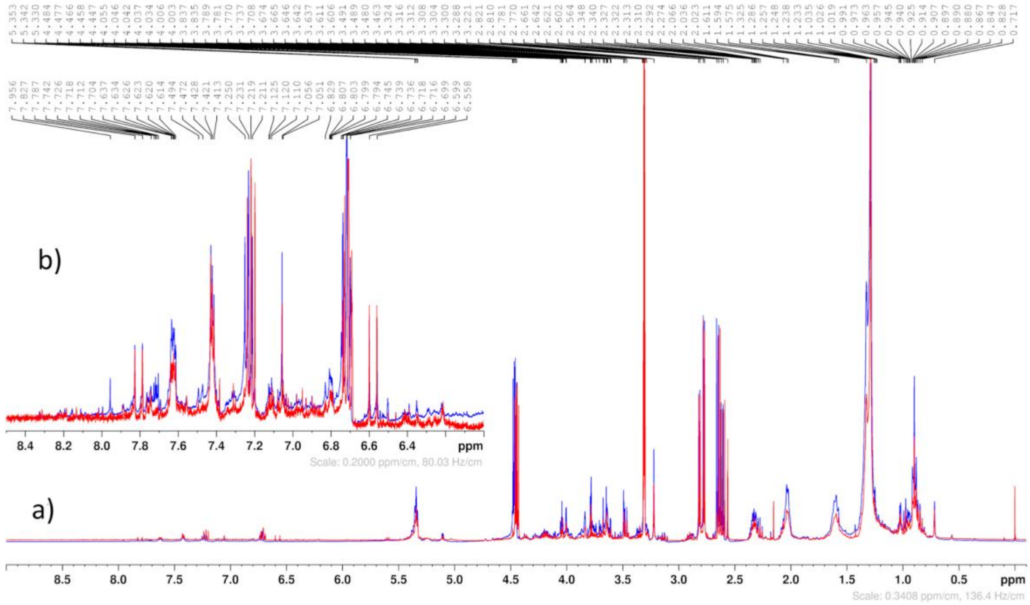

Figure S3. (a) ^{1}H NMR spectra of AHBAc (blue) and AHKAc (red) recorded in MeOD-*d*$_4$. **(b)** Expanded region, δ 6.0–8.5. Spectra acquired on a 400 MHz spectrometer for proton.

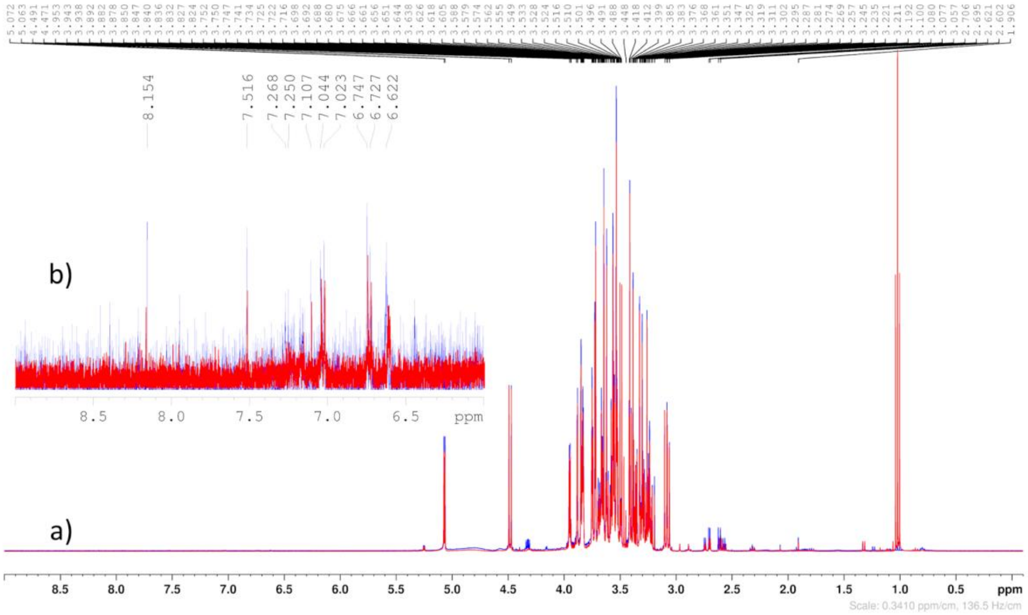

Figure S4. (a) ^{1}H NMR spectra of AHBAq (blue) and AHKAq (red) recorded in MeOD-*d*$_4$. **(b)** Expanded region, δ 6.0–9.0. Spectra acquired on a 400 MHz spectrometer for proton.

HPLC-ESI-QTOF-MS/MS

Chromatogram

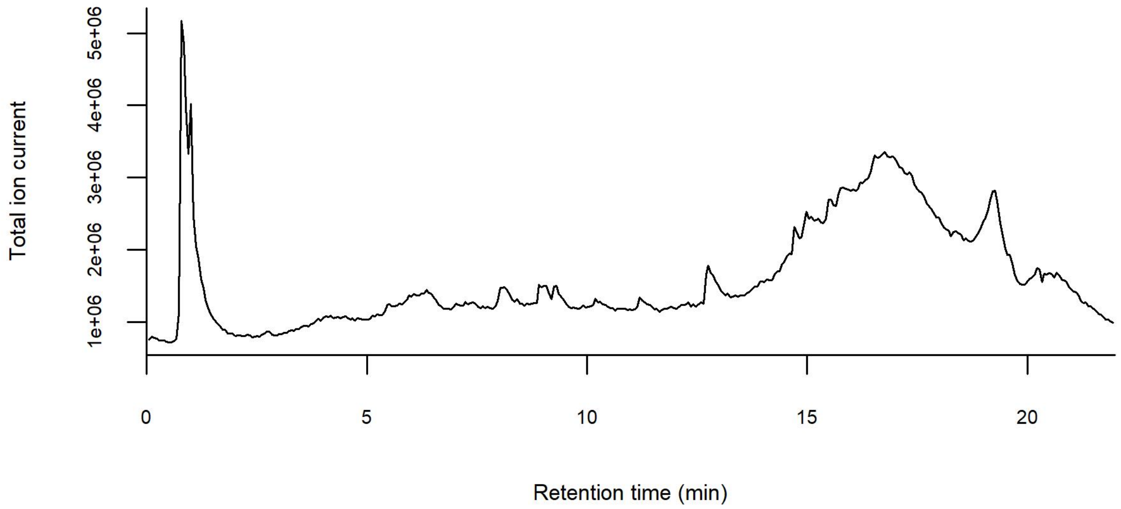

Figure S5. HPLC–ESI–QTOF–MS/MS base peak chromatogram (BPC) of the crude ethanolic extract from mixed pseudofruits of *A. humile* and *A. occidentale* from Brazlândia (AHB), acquired in positive ionization mode.

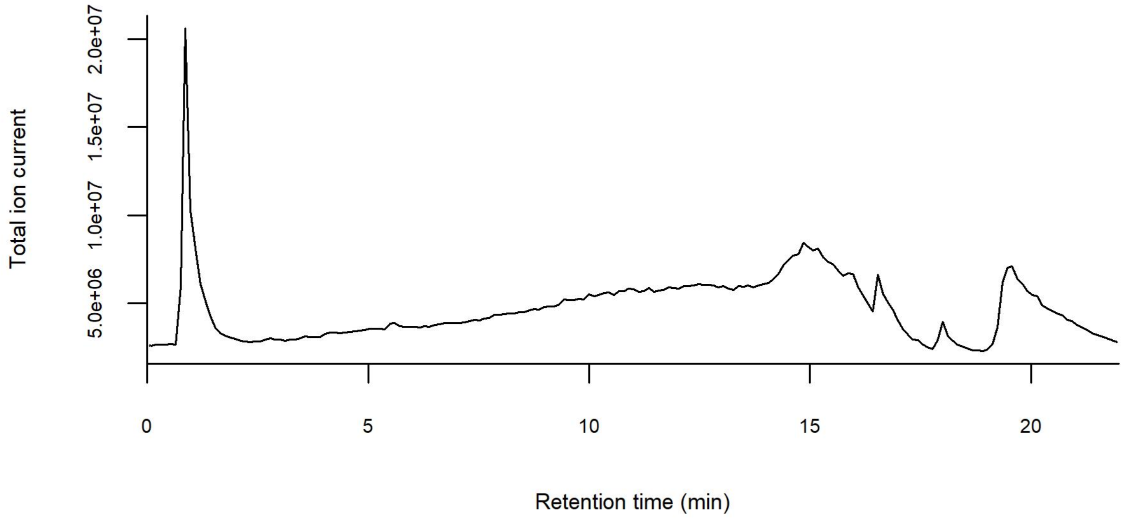

Figure S6. HPLC–ESI–QTOF–MS/MS base peak chromatogram (BPC) of the crude ethanolic extract from mixed pseudofruits of *A. humile* and *A. occidentale* from Brazlândia (AHB), acquired in negative ionization mode.

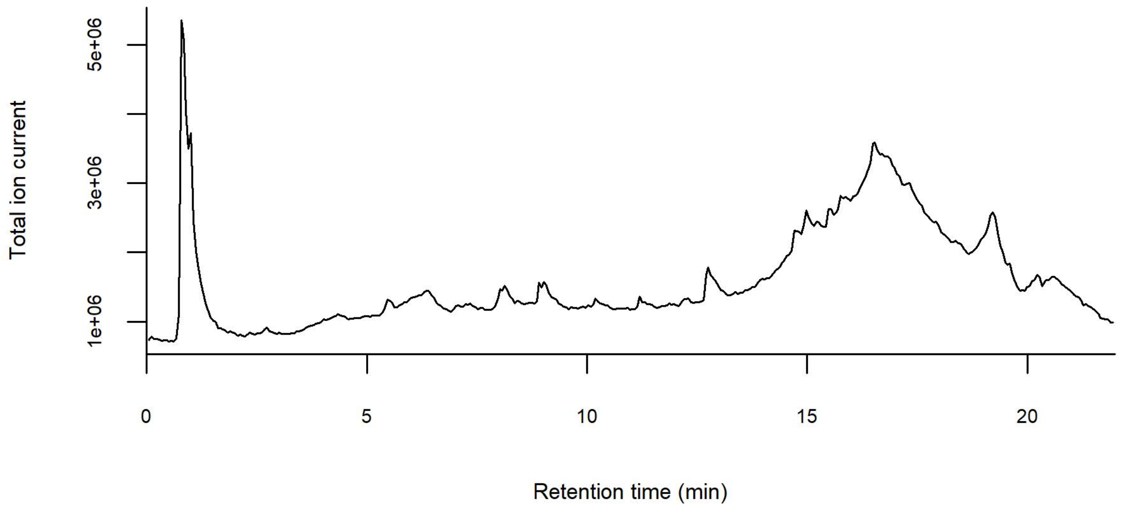

Figure S7. HPLC–ESI–QTOF–MS/MS base peak chromatogram (BPC) of the crude ethanolic extract from mixed pseudofruits of *A. humile* and *A. occidentale* from Kalunga community (AHK), acquired in positive ionization mode.

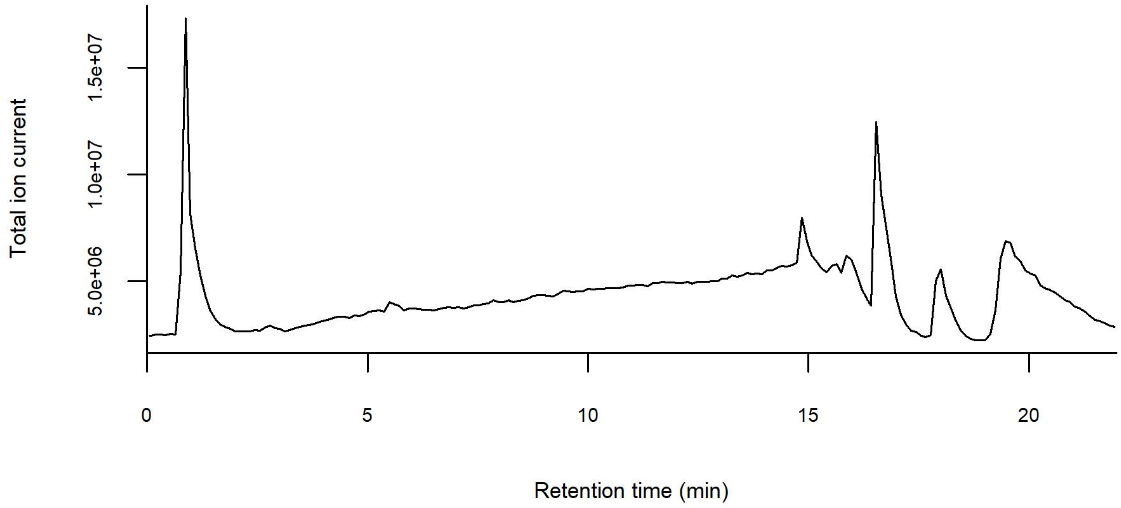

Figure S8. HPLC–ESI–QTOF–MS/MS base peak chromatogram (BPC) of the crude ethanolic extract from mixed pseudofruits of *A. humile* and *A. occidentale* from Kalunga community (AHK), acquired in negative ionization mode.

Mass spectra of the annotated compounds

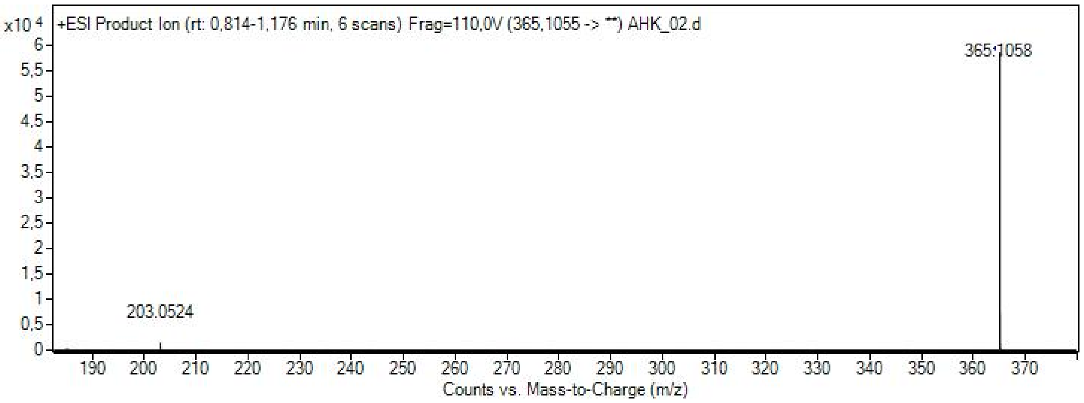

Figure S9. Mass spectrum of disaccharide **(2)** obtained in positive ionization mode (ESI+) from AHB and AHK ethanolic extracts.

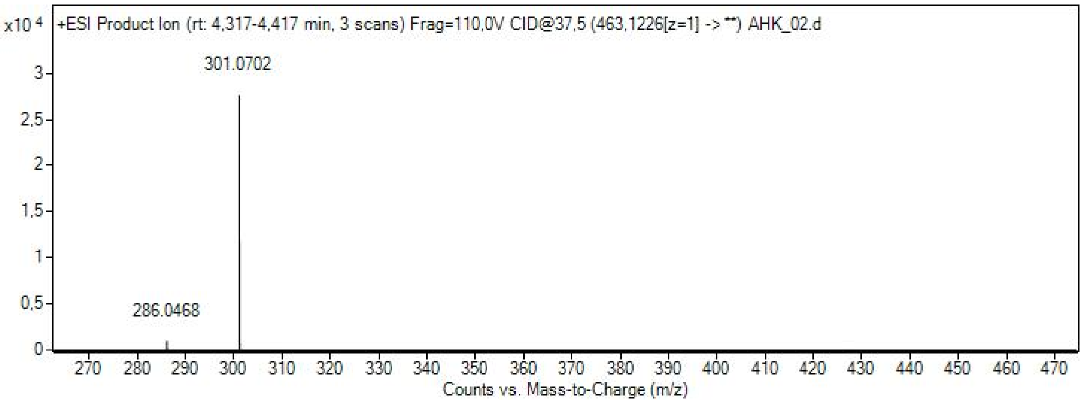

Figure S10. Mass spectrum of peonidin *O*-hexoside **(3)** obtained in positive ionization mode (ESI+) from AHB and AHK ethanolic extracts.

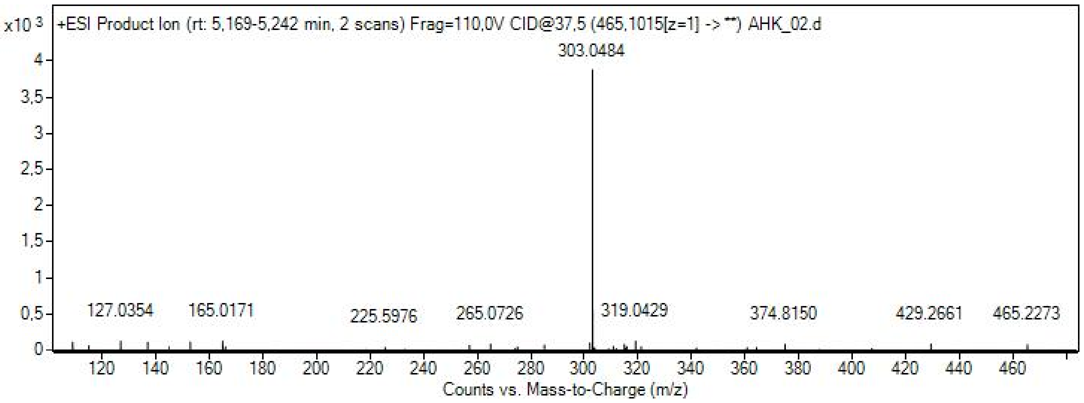

Figure S11. Mass spectrum of quercetin *O*-hexoside **(4)** obtained in positive ionization mode (ESI+), detected only in AHK ethanolic extract.

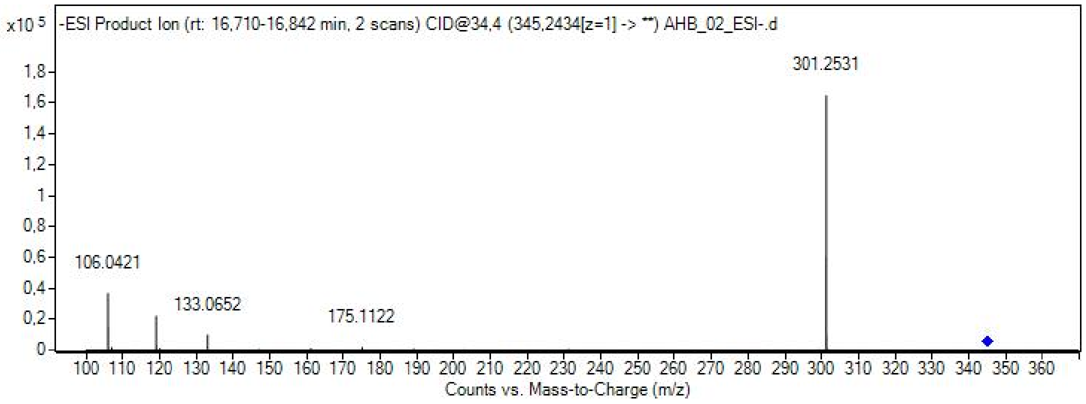

Figure S12. Mass spectrum of ginkgolic acid (15:1) **(5)** obtained in negative ionization mode (ESI-) from AHB and AHK ethanolic extracts.

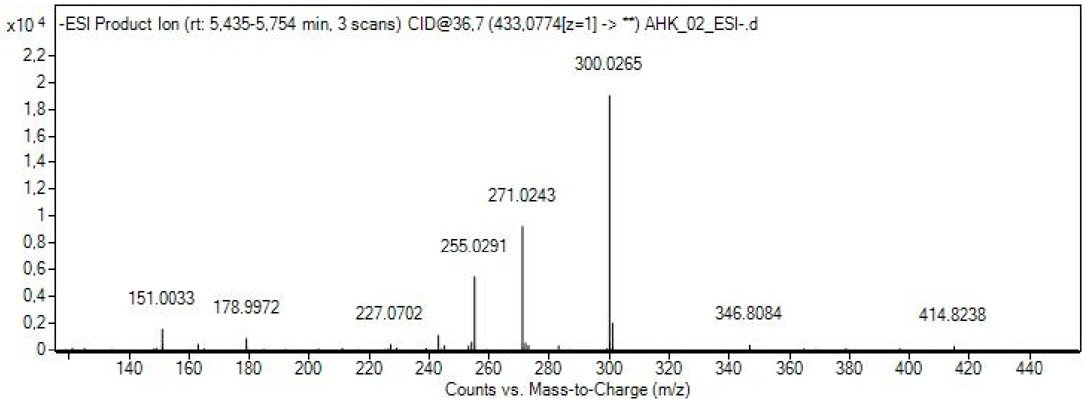

Figure S13. Mass spectrum of quercetin *O*-pentoside **(6)** obtained in negative ionization mode (ESI-), detected only in AHK ethanolic extract.

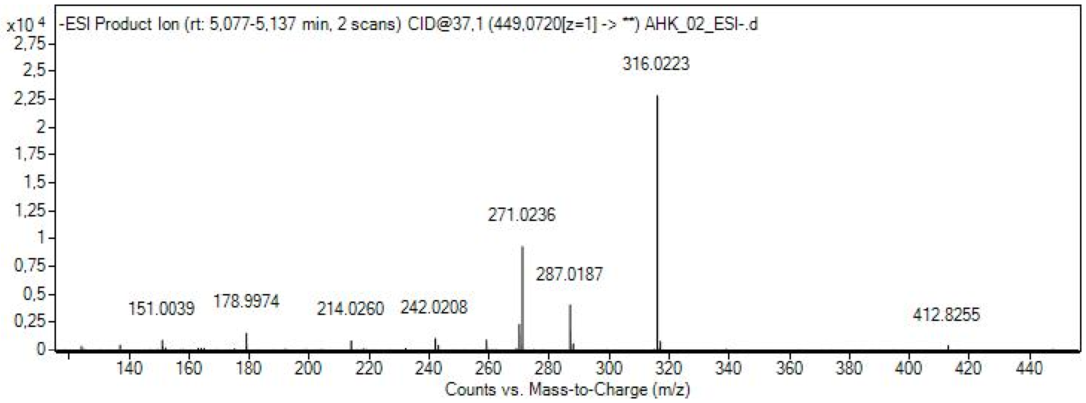

Figure S14. Mass spectrum of myricetin *O*-pentoside **(7)** obtained in negative ionization mode (ESI-), detected only in AHK ethanolic extract.

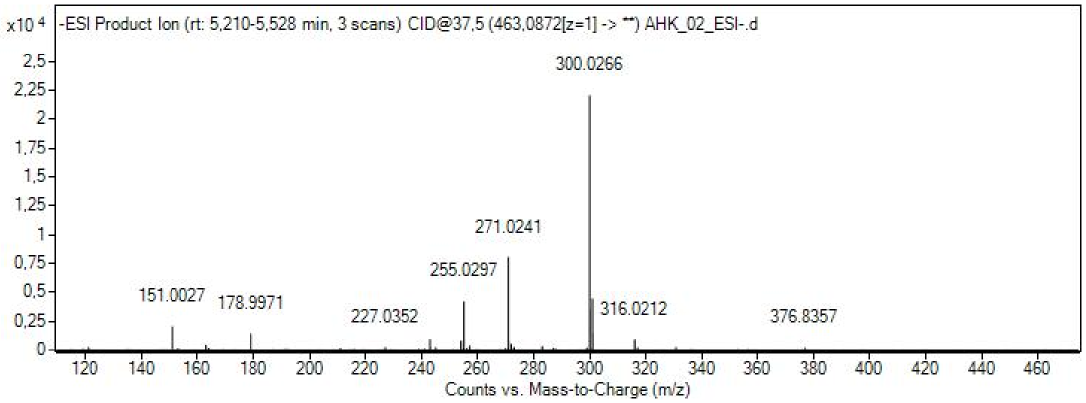

Figure S15. Mass spectrum of quercetin *O*-hexoside **(8)** obtained in negative ionization mode (ESI-) from AHB and AHK ethanolic extracts.

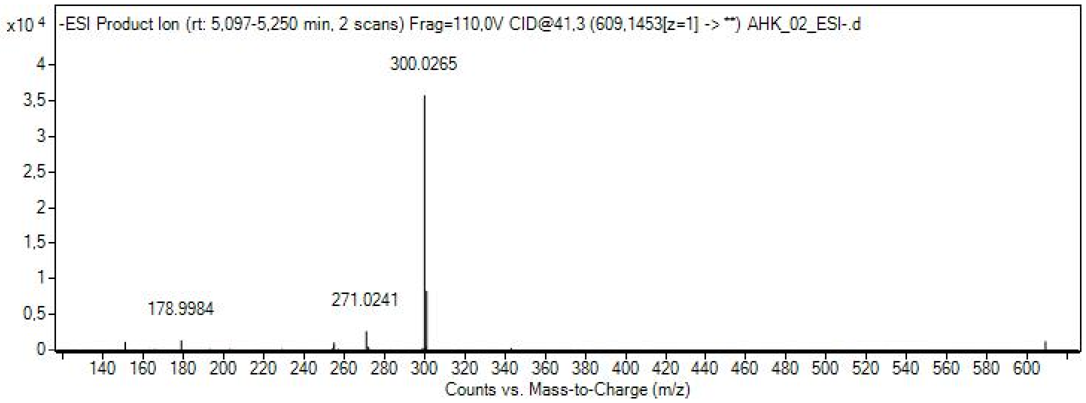

Figure S16. Mass spectrum of quercetin *O*-hexosyl-deoxyhexoside **(9)** obtained in negative ionization mode (ESI-) from AHB and AHK ethanolic extracts.

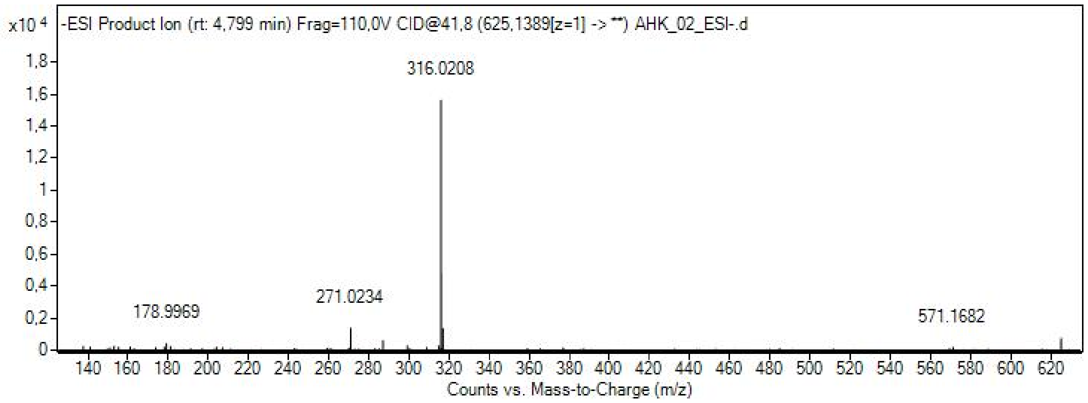

Figure S17. Mass spectrum of myricetin *O*-hexosyl-deoxyhexoside **(10)** obtained in negative ionization mode (ESI-) from AHB and AHK ethanolic extracts.

Proposed fragmentation mechanisms for dereplicated compounds

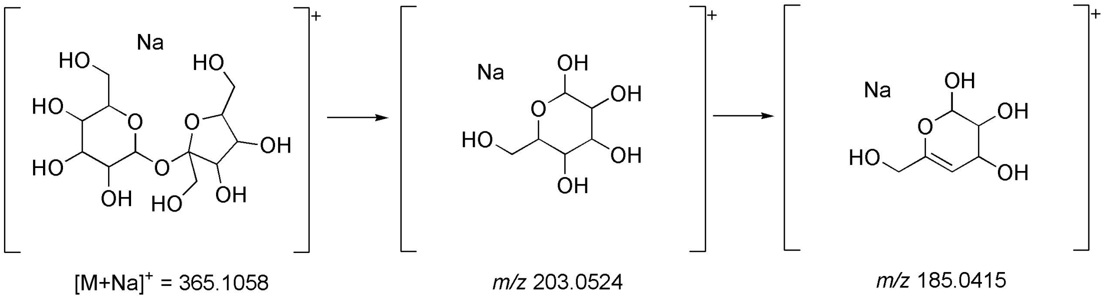

Figure S18. Proposed MS/MS fragmentation pathway for the disaccharide **(2)** annotated in positive ionization mode.

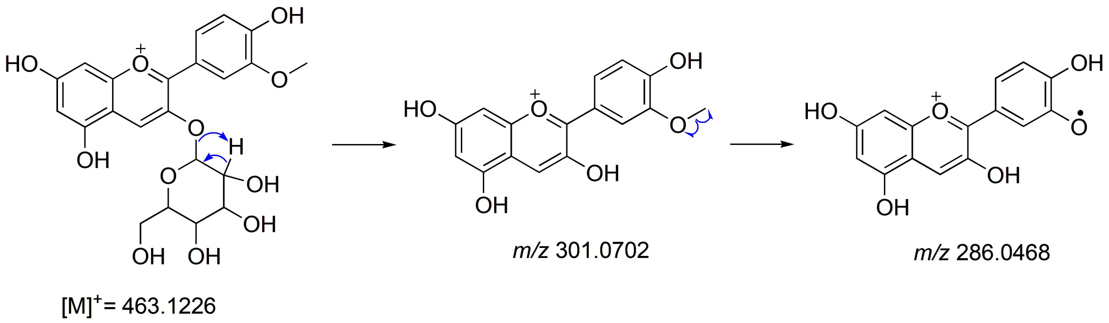

Figure S19. Proposed MS/MS fragmentation pathway for peonidin *O*-hexoside **(3)** in positive ionization mode.

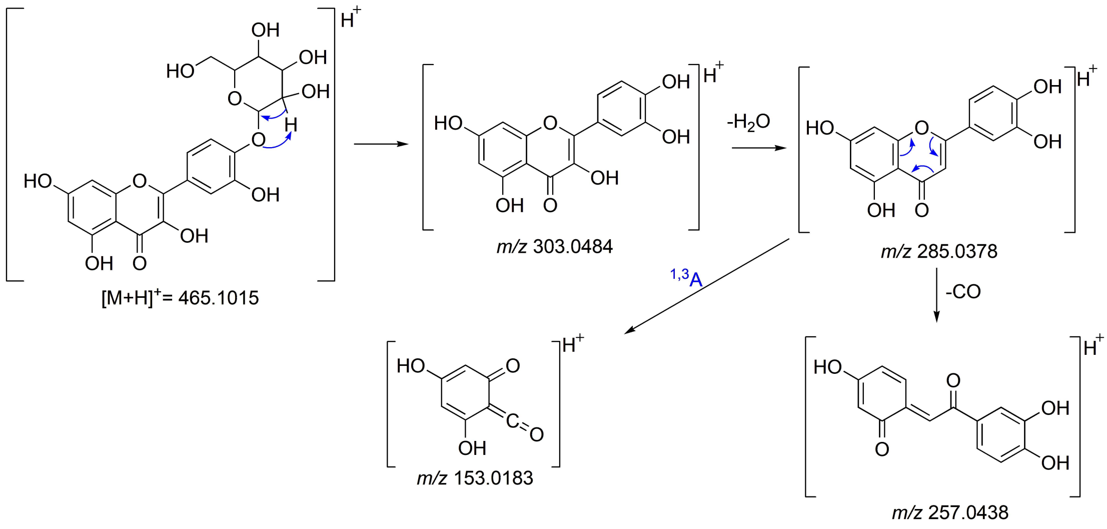

Figure S20. Proposed MS/MS fragmentation pathway for quercetin *O*-hexoside **(4)** in negative ionization mode.

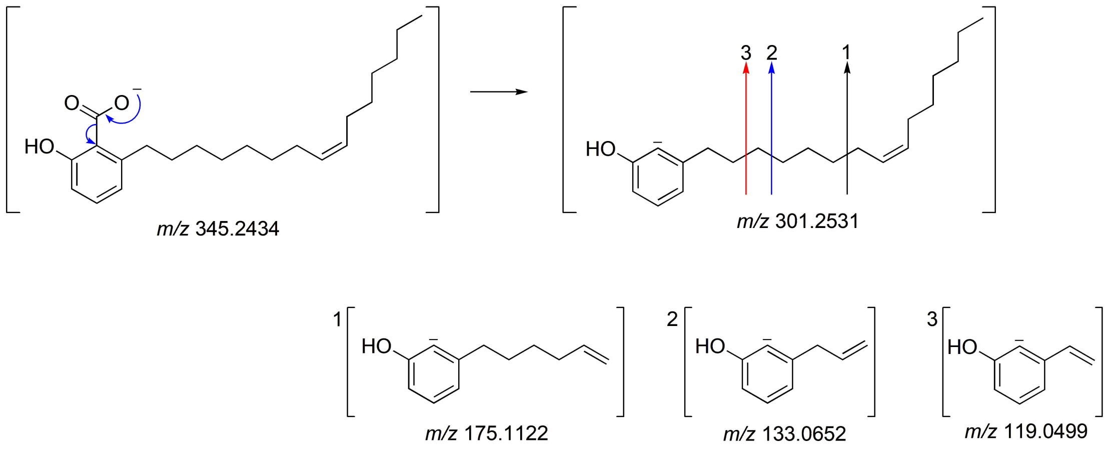

Figure S21. Proposed MS/MS fragmentation pathway for ginkgolic acid (15:1) **(5)** in negative ionization mode.

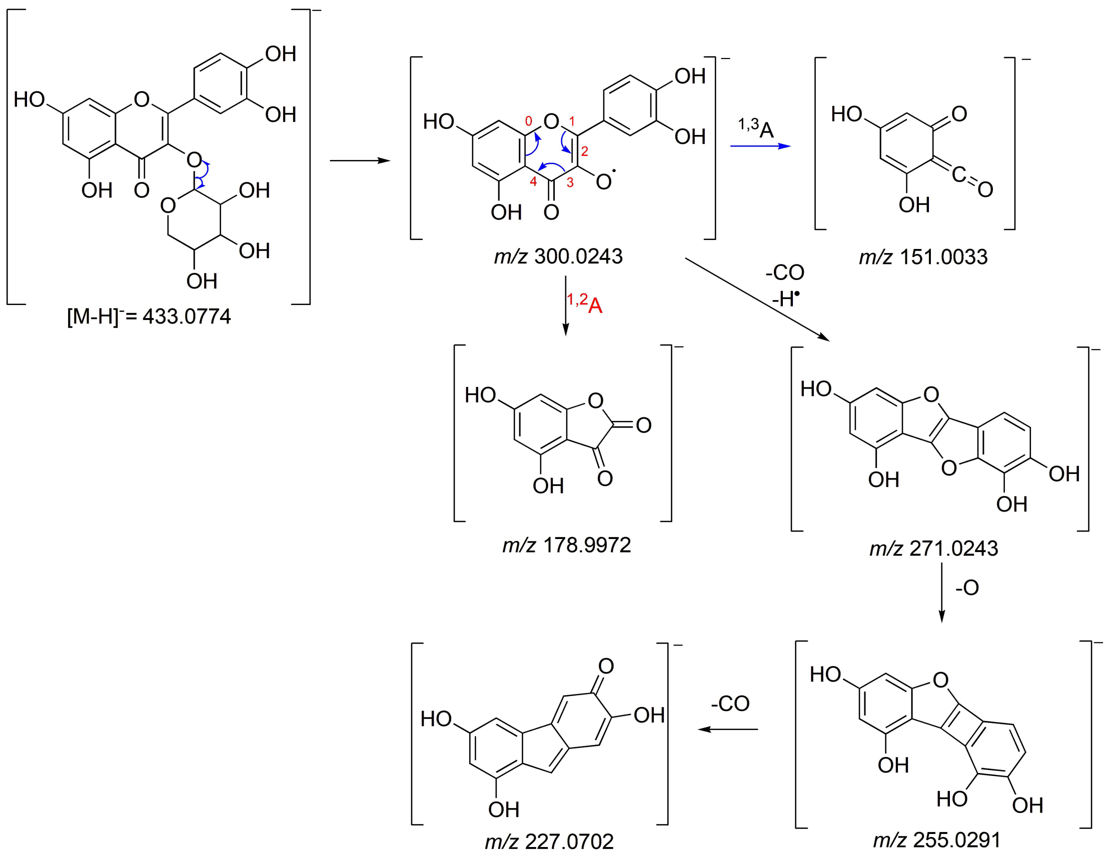

Figure S22. Proposed MS/MS fragmentation pathway for quercetin *O*-pentoside **(6)** in negative ionization mode.

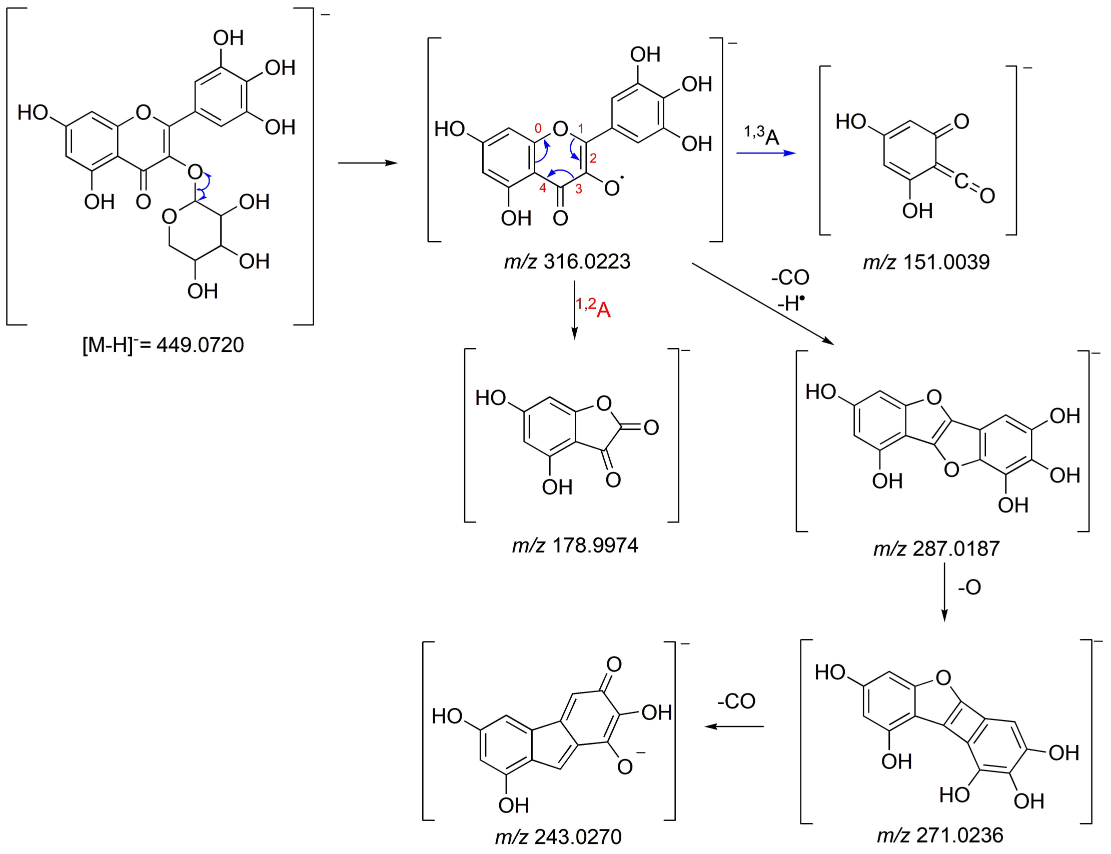

Figure S23. Proposed MS/MS fragmentation pathway for myricetin *O*-pentoside **(7)** in positive ionization mode.

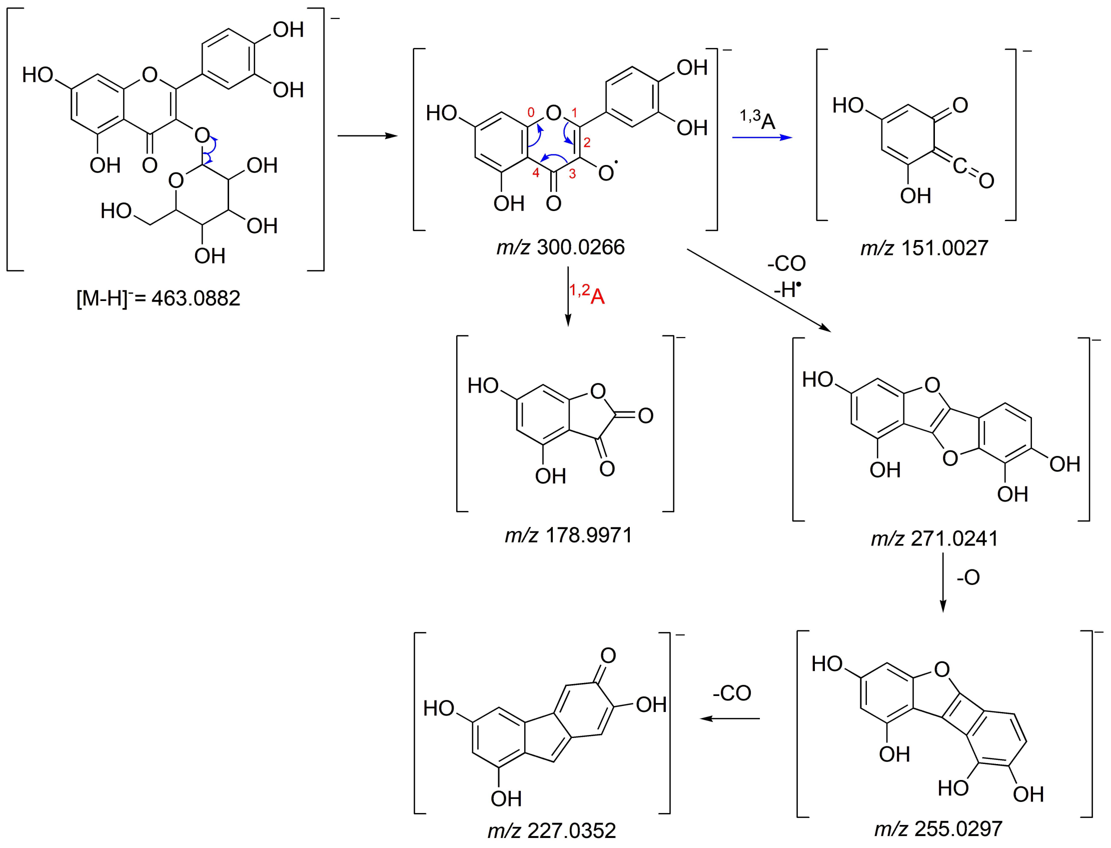

Figure S24. Proposed MS/MS fragmentation pathway for quercetin *O*-hexoside **(8)** in negative ionization mode.

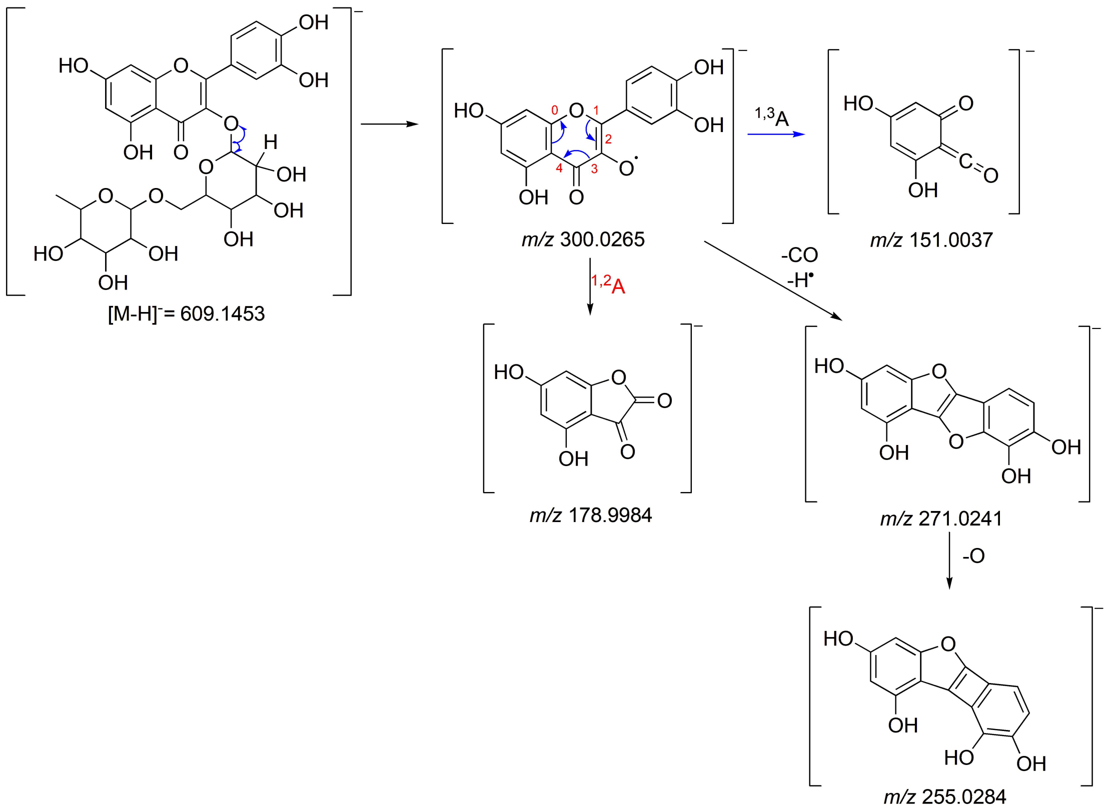

Figure S25. Proposed MS/MS fragmentation pathway for quercetin *O*-hexosyl-deoxyhexoside **(9)** in negative ionization mode.

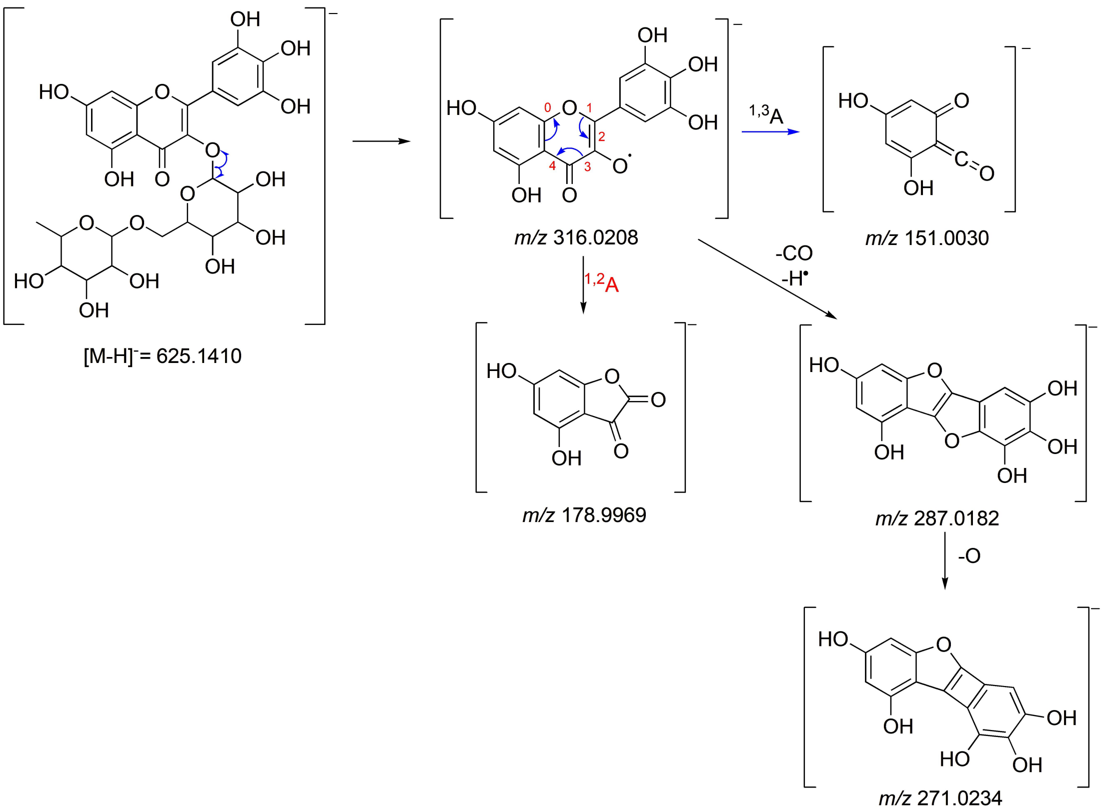

Figure S26. Proposed MS/MS fragmentation pathway for myricetin *O*-hexosyl-deoxyhexoside **(10)** in negative ionization mode.

Molecular Networks

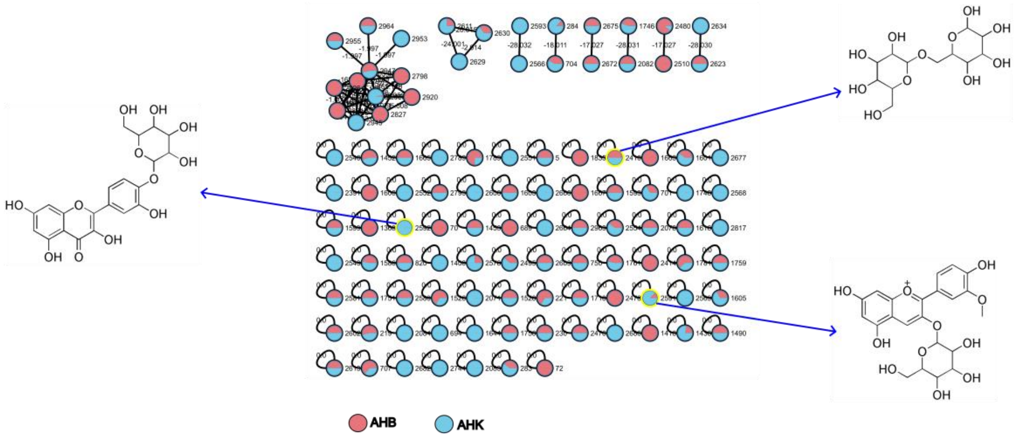

Figure S27. Molecular network from classical analysis generated on the GNPS platform for AHB (red) and AHK (blue) ethanolic extracts in positive ionization mode,

including annotated compounds. Nodes corresponding to annotated features are highlighted in yellow.

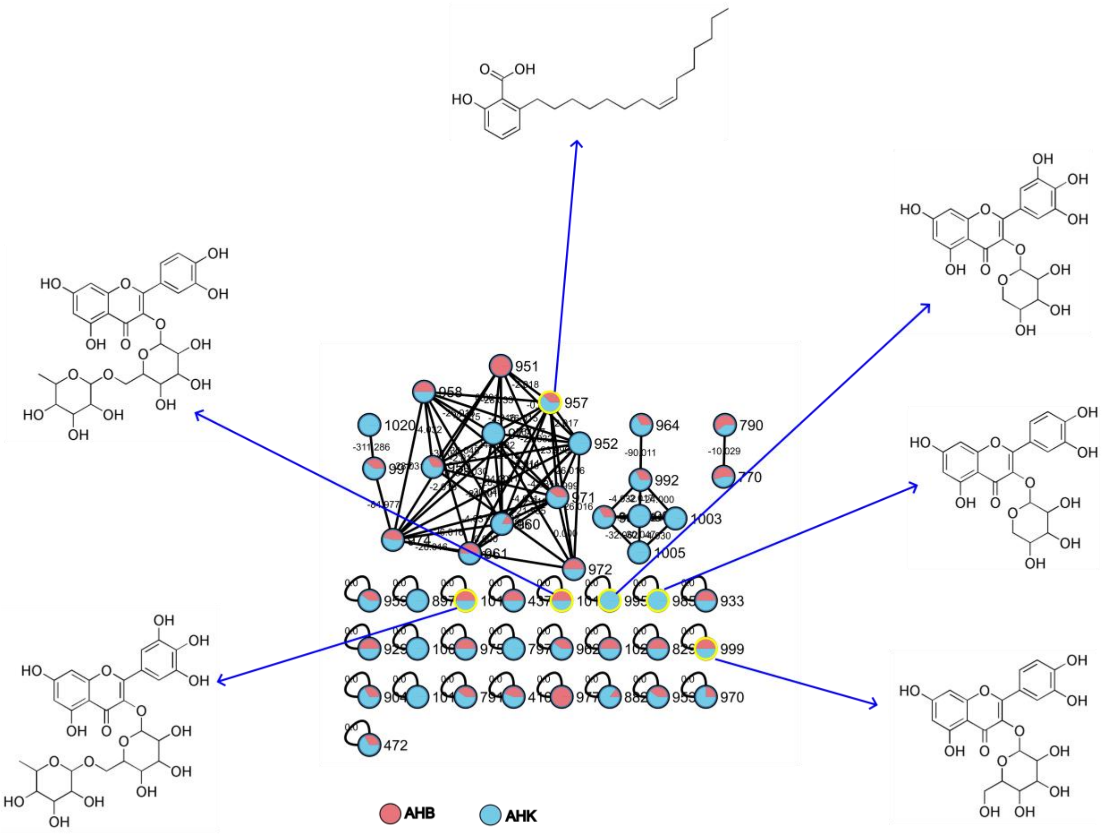

Figure S28. Molecular network from classical analysis generated on the GNPS platform for AHB (red) and AHK (blue) ethanolic extracts in negative ionization mode, including annotated compounds. Nodes corresponding to annotated features are highlighted in yellow.

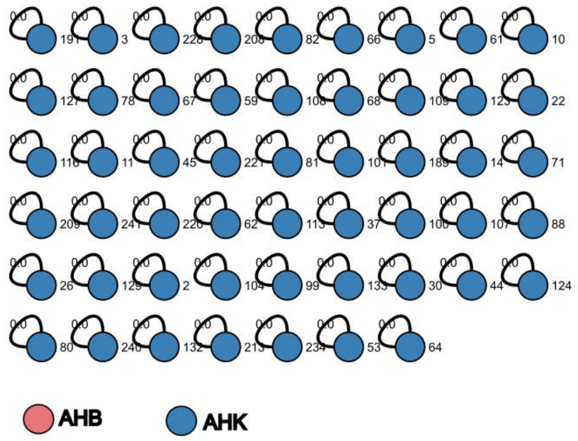

Figure S29. Feature-based molecular network (FBMN) generated on the GNPS platform for AHB (red) and AHK (blue) ethanolic extracts in positive ionization mode. No annotated compounds were detected.

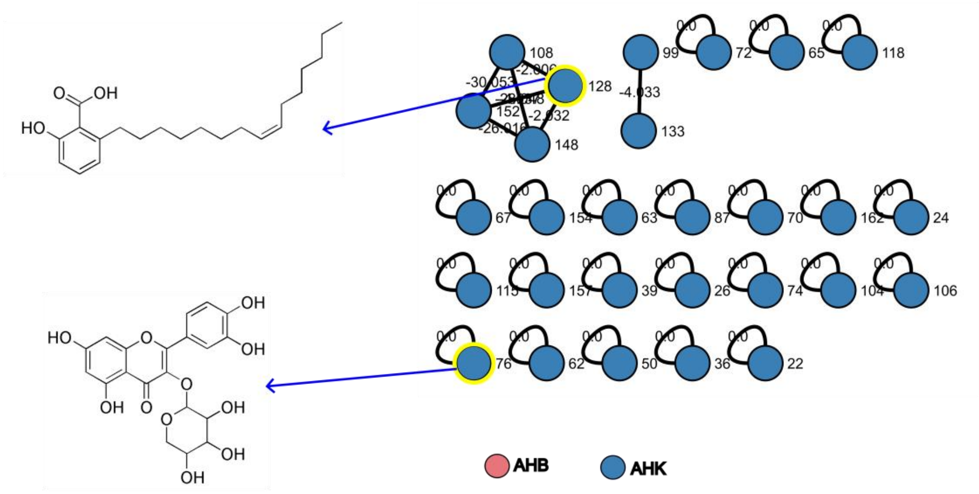

Figure S30. Feature-based molecular network (FBMN) generated on the GNPS platform for AHB (red) and AHK (blue) ethanolic extracts in negative ionization mode, including annotated compounds. Nodes corresponding to annotated features are highlighted in yellow.

Thermal Stability (TG-DTG)

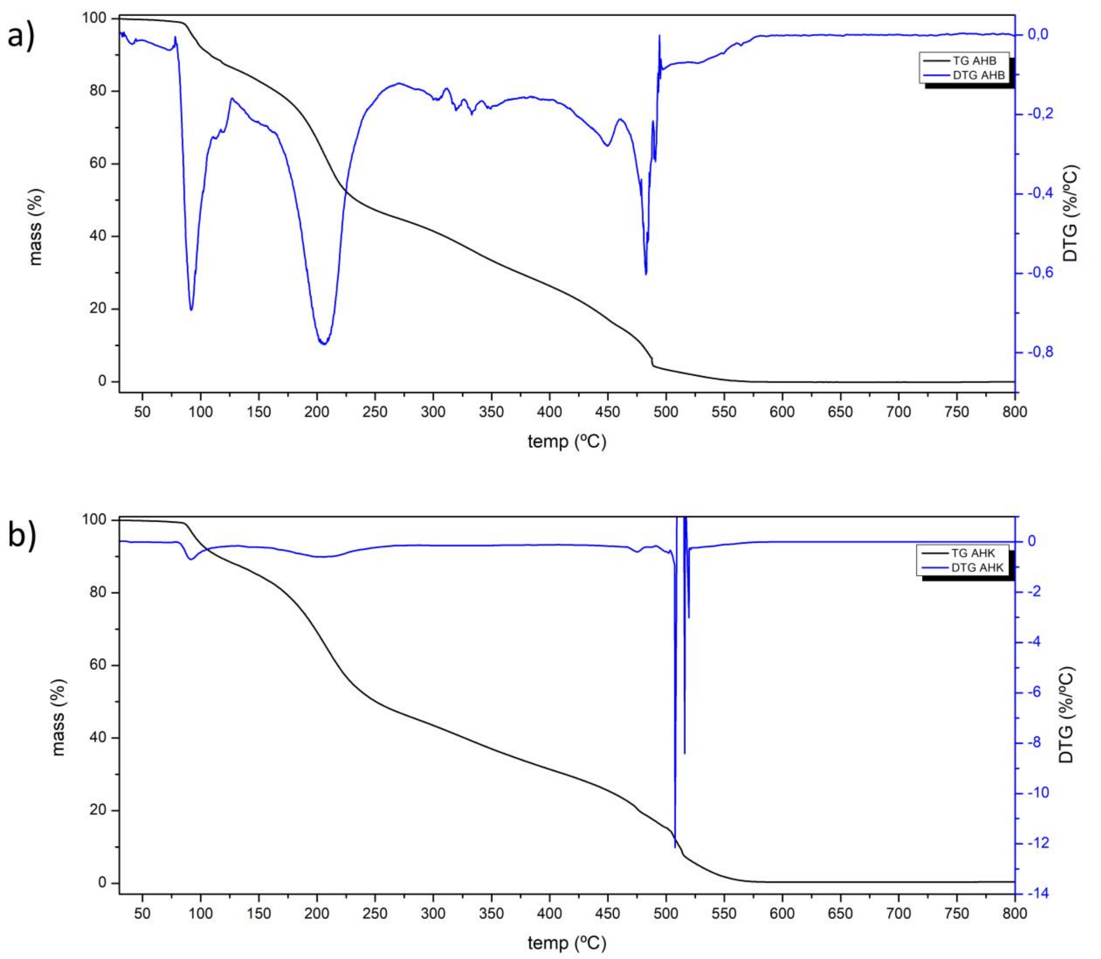

Figure S31. TG-DTG curves for AHB and AHK crude ethanolic extracts. TG curves (black) show mass loss with increasing temperature, whereas DTG curves (blue) indicate the onset and end of each thermal event. **(a)** AHB – pseudofruits from Brazlândia, **(b)** AHK – pseudofruits from the Kalunga community.

Table S1. Thermal events, mass losses, and residues for AHB and AHK crude ethanolic extracts.

Sample	Steps	ΔT (°C)	DTG peak (°C)	Δm (%)	Residue (%)
**AHB**	1st	31.29 - 126.79	91.82	13.46	0

	2nd	126.79 - 270.43	205.53	41.60	
	3rd	270.43 - 390.38	333.00	17.23	
	4th	390.38 - 460.02	449.71	12.67	
	5th	460.02 - 580.73	482.45	15.04	
**AHK**	1st	33.49 -120.01	91.61	12.57	0
	3rd	120.01 - 268.74	205.84	42.35	
	4th	268.74 - 414.99	333.97	19.76	
	5th	414.99 - 495.71	475.11	16.23	
	6th	495.71 - 587.63	507.68	9.09	

Photochemical study

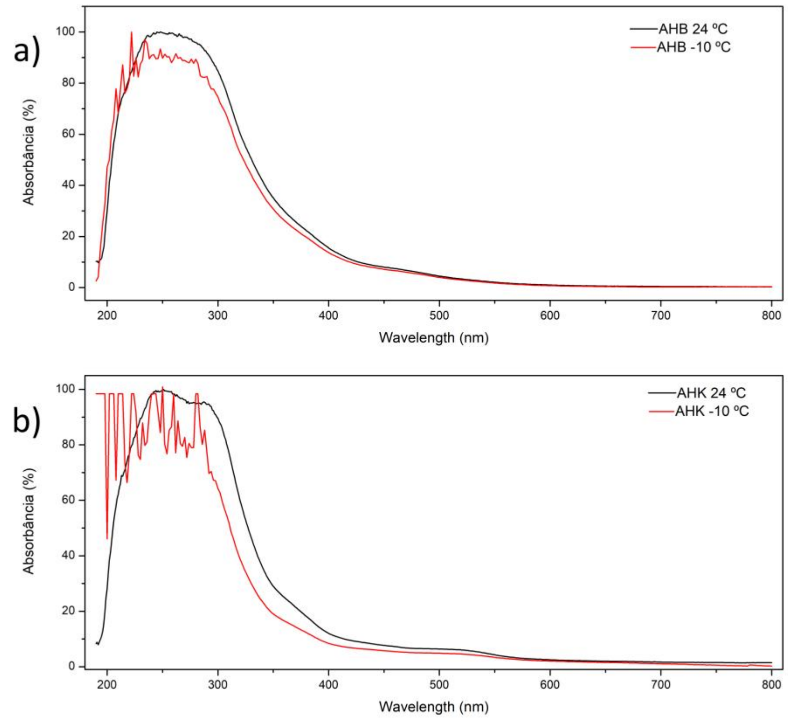

Figure S32. UV–Vis absorption spectra of AHB **(a)** and AHK **(b)** extracts recorded at -10 and 24 °C over the wavelength range 190–800 nm.

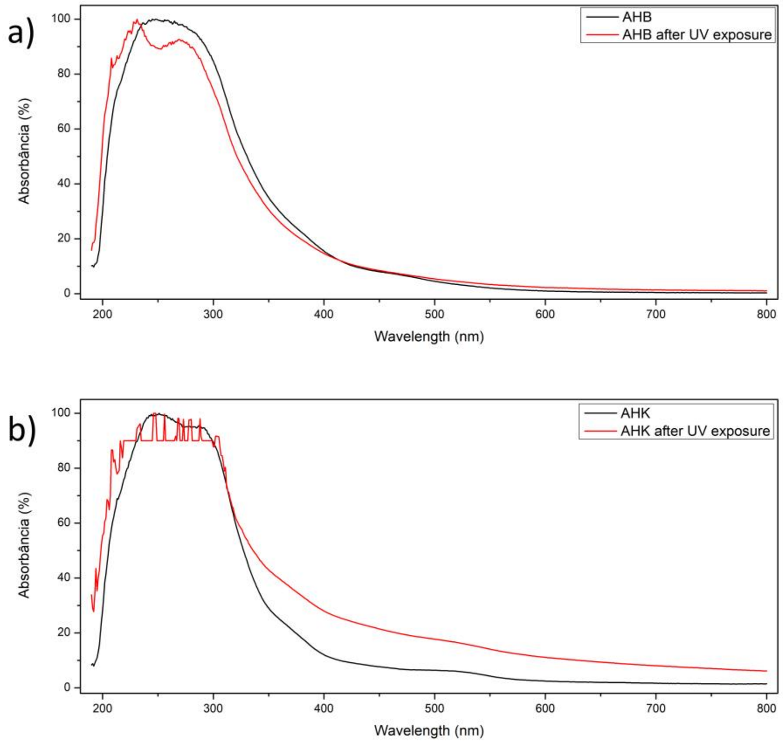

Figure S33. UV–Vis absorption spectra of AHB **(a)** and AHK (b) extracts before and after exposure to ultraviolet radiation at wavelengths 254 and 363 nm.
